# Multiwaves, breathers, lump and other solutions for the Heimburg model in biomembranes and nerves

**DOI:** 10.1038/s41598-024-60689-0

**Published:** 2024-05-03

**Authors:** Dilber Uzun Ozsahin, Baboucarr Ceesay, Muhammad Zafarullah baber, Nauman Ahmed, Ali Raza, Muhammad Rafiq, Hijaz Ahmad, Fuad A. Awwad, Emad A. A. Ismail

**Affiliations:** 1 Department of Medical Diagnostic Imaging, College of Health Sciences, Sharjah University, Sharjah, United Arab Emirates; 2 Research Institute for Medical and Health Sciences, University of Sharjah, Sharjah, United Arab Emirates; 3 Mathematics and Statistics Department, The University of Lahore, Lahore, Pakistan; 4 Mathematics Unit, The University of The Gambia, Sere Kunda, The Gambia; 5 Department of Mathematics, Govt. Maulana Zafar Ali Khan Graduate College Wazirabad, Punjab Higher Education Department (PHED), Lahore, 54000 Pakistan; 6 Department of Mathematics, Faculty of Science and Technology, University of Central Punjab, Lahore, Pakistan; 7 Section of Mathematics, International Telematic University Uninettuno, Corso Vittorio Emanuele II, 39,00186 Roma, Italy; 8 Near East University, Operational Research Center in Healthcare, TRNC Mersin 10, Nicosia, 99138, Turkey; 9 Department of Quantitative analysis, College of Business Administration, King Saud University, P.O. Box 71115, 11587 Riyadh, Saudi Arabia; 10 Department of Computer Science and Mathematics, Lebanese American University, Beirut, Lebanon; 11 Center for Applied Mathematics and Bioinformatics, Gulf University for Science and Technology, Mishref, Kuwait

**Keywords:** Heimburg model, Hirota bilinear method, Soliton solutions, Physical representation, Computational biology and bioinformatics, Mathematics and computing

## Abstract

In this manuscript, a mathematical model known as the Heimburg model is investigated analytically to get the soliton solutions. Both biomembranes and nerves can be studied using this model. The cell membrane’s lipid bilayer is regarded by the model as a substance that experiences phase transitions. It implies that the membrane responds to electrical disruptions in a nonlinear way. The importance of ionic conductance in nerve impulse propagation is shown by Heimburg’s model. The dynamics of the electromechanical pulse in a nerve are analytically investigated using the Hirota Bilinear method. The various types of solitons are investigates, such as homoclinic breather waves, interaction via double exponents, lump waves, multi-wave, mixed type solutions, and periodic cross kink solutions. The electromechanical pulse’s ensuing three-dimensional and contour shapes offer crucial insight into how nerves function and may one day be used in medicine and the biological sciences. Our grasp of soliton dynamics is improved by this research, which also opens up new directions for biomedical investigation and medical developments. A few 3D and contour profiles have also been created for new solutions, and interaction behaviors have also been shown.

## Introduction

Advances in nonlinear partial differential equations (NLPDEs), a powerful tool for many interdisciplinary investigations, have made it possible to investigate complex physical phenomena in disciplines like fluid science, control theory, hydrodynamics, geochemistry, optical science, and plasma^1,2^. In biological systems, soliton production and propagation in neurons and biomembranes is crucial^3,4^. Accurate NLPDE solutions are critical for comprehending the complex mechanisms that govern these procedures.

The recent development of novel techniques for obtaining soliton solutions from NLPDEs has significantly improved our ability to recognize and investigate these occurrences. Finding exact soliton solutions proved to be an extremely successful application of the Hirota bilinear technique^5,6^. Among the applications of this method that have proved successful include the analysis of nonlinear Schrödinger equations, integrable systems, and optical fibers^7,8^. Regarding the mechanical systems that support nerves and biomembranes, the Heimburg model offers essential details. The nerve axon is portrayed in the model as a cylinder-shaped biomembrane that changes from a fluid to a gel structure at a specific temperature below average. The soliton dynamics in this framework provide crucial information regarding the nature of nerve impulses, which makes it crucial to understand.

The Hirota bilinear technique^9,10^ is utilized in this investigation to evaluate the soliton production and propagation in biomembranes and neurons employing the Heimburg model. Using this technique, we can obtain precise soliton solutions and learn more about how electromechanical pulses behave differently within nerves. This analytical method illuminates the basic concepts that underlie biological systems by studying the intricate interactions between biomembranes and solitons. By combining the power of the Hirota bilinear technique with the understanding gained from the Heimburg model, this study contributes to a clearer understanding of the complicated dynamics of soliton occurrences in biological systems. The findings might influence a variety of fields, such as biophysics, neuroscience, and bioengineering, and they might hasten the development of therapeutic interventions and bio-inspired technology.

The Heimburg model is an integrable differential equation, there is no general technique to solve these equation. Zhang et al. proposed the symbol calculation method based on neural networks to obtained the exact analytical solutions for the NLPDEs. The Bilinear residual network method is used to obtained the exactly explicit solutions for the nonlinear evolution equations^11^. For the first time bilinear neural network model is used to get the exact analytical solution for the reduced p-gBKP equation^12^, the different types of soliton solutions are also constructed by using this technique for the (3+ 1)-dimensional Jimbo-Miwa equation^13^. The new test functions Fractal solitons, arbitrary function solutions, exact periodic wave, breathers, generalized lump solutions, classical lump solutions and rogue waves are constructed^14–16^, and the interference wave and the bright and dark soliton are also constructed via bilinear residual network method^17,18^.

Khatun, et al., worked on the couple modified equal-width and Boussinesq equations constructed the soliton solutions by using the Sine-Gordon expansion^19^. Arefin, et al., investigated the closed form travelling wave solution for the non-linear evolution equations using the two-variable $(G'/G, 1/G)$-expansion method^20^. Zaman, et al., explored soliton wave propagation for the nonlinear coupled type Boussinesq-Burger (BB) and coupled type Boussinesq equations via extended tanh-function method^21^. Pan, et al., worked on the derivative nonlinear Schrödinger equation to construct the optical soliton solutions by using the extended modified auxiliary equation mapping technique^22^. Seadawy et al., used the extended modified auxiliary equation mapping to explored the optical solitons for the perturbed nonlinear fractional Schrödinger equation^23^ and the integrable improved perturbed nonlinear Schrödinger equation with type of Kerr law nonlinearity^24,25^. Cheemaa et al., analyzed the soliton solutions for the nonlinear modified Korteweg-de Vries equation using the auxiliary equation mapping method^26^.

A mathematical method utilised in the study of nonlinear systems and soliton theory is the Hirota bilinear transformation. It was developed by Ryogo Hirota to explore soliton of nonlinear equations, particularly PDEs that admit soliton solutions - localised, stable, and often interacting wave-like structures. The Hirota bilinear transformation’s main goal is to express NLPDEs in a bilinear form. This bilinear form facilitates the investigation of the underlying dynamics and makes it possible to develop multi-soliton solutions. Once the soliton equation is expressed in bilinear form, solutions can be obtained by solving the resulting system of bilinear equations. The bilinear form facilitates the study of multi-soliton solutions, their interactions, and the underlying dynamics of the original soliton equation. The solutions obtained through the Hirota bilinear transformation provide insights into the behavior of solitons in the system described by the original equation. These solutions often exhibit interesting phenomena such as soliton collisions, fusion, and fission. In travelling wave theory, the application of asymptotic techniques helps to fully comprehend the dynamics of solitons by capturing their leading-order behaviour. This method also forms the basis for numerical simulations, which direct and validate computational studies on solitons in NLPDEs. In general, travelling wave theory shows to be an effective and adaptable method for understanding the complex nature of soliton occurrences in a range of nonlinear systems. So, under considered method is more effective our the other analytical techniques because the special types of solitons are generated. This approach is provided us the special types of soliton solutions such as, breather wave, Lump wave, multi-wave, mixed wave, M-shape, rough wave, one kink, two kink, periodic cross kink and many others. But method are provided us only hyperbolic, trigonometric and rational wave solutions.

But in this study we use hirota bilinear method which is efficient technique that will provided us the different form of solitons like, breather wave, lump wave, multiwave, M-shapes and many other interactions. The different types of soliton solutions for the Heimburg model. These solutions have many applications in biomembranes and nerves can be studied using this model. The Hirota bilinear transformation is used to construct the homoclinic breather waves, interaction via double exponents, lump waves, multi-wave, mixed type solutions, and periodic cross kink solutions. These solution have there significance in the dynamical study of biomembranes and nerves which also opens up new directions for biomedical investigation and medical developments. The soliton are plotted in 3D and contour profiles for new solutions, and interaction behaviors have also been shown. There plots are show the Breather waves, lump waves, multiple wave, periodic cross kink solutions. These plots have the physical significance in the energy propagation patterns inside the biomembranes. It’s crucial to remember, nevertheless, that generating real graphical representations could call for specific tools and software. A breather wave is an oscillating or pulsing localized disturbance that moves through the membrane. Seek for a graph that illustrates a spike or disturbance that occurs at a specific point along the membrane and then vanishes. Over time, the wave’s amplitude could change. A concentrated, non-dispersive energy package passing across the membrane is represented as a lump wave. Imagine a wave that propagates through the membrane without greatly expanding or altering form, all the while maintaining its amplitude and shape. The coexistence of many waves in the membrane at various frequencies and amplitudes is referred to as multiple waves. Look for a graph that shows several waveforms interacting with one another inside the membrane, each with a different frequency and amplitude. These plot have the significance in the theoretical and experiential study of nonlinear Heimburg model.

The nonlinear Heimburg model will be introduced in “An overview of the model” to provide a theoretical basis for our study. “An overview of the method” will describe the Hirota bilinear method, which we will use to conduct our analysis. “Application of the method on the Heimburg model” will apply the Hirota bilinear method to the Heimburg model, extracting exact soliton solutions, and examining their properties. “Graphical presentations” will deal with the findings by presenting them in graphical illustrations and in “Conclusion” we conclude on our findings.

## An overview of the model

Numerous mathematical models have been used to examine the propagation of electrical signals in nerve axons in great detail^27–29^. Action potential dynamics have been better understood thanks to the Hodgkin-Huxley model^30^, which is based on the reaction-diffusion equation. The more straightforward model put forth by FitzHugh and Nagumo has also provided a more manageable framework for studying pulse propagation^30^. These models, however, do not take into consideration how nerve conduction works mechanically. We offer the Heimburg model to fill up this knowledge gap, which integrates electrical and mechanical dynamics to provide a more comprehensive comprehension of sound transmission in nerve axons. In the Heimburg model, proteins are viewed as resistors and membranes as capacitors in an electrical circuit that represents the nerve axon. With the use of this conceptualization, we may define the voltage fluctuation across the neuron membrane as a propagating action potential^31^. Along the nerve axon, a voltage pulse is created by the ion currents the membrane produces^31^. We take into account lateral density excitations within a one-dimensional cylindrical nerve axon to take the mechanical elements into account. The following equation can be used to describe how sound propagates via nerve axons without dispersion^1^

1 $$\begin{aligned} \frac{{\partial ^2 \Delta \rho ^A}}{{\partial \tau ^2}} = \frac{\partial }{\partial z}\left( c^2 \frac{{\partial \Delta \rho ^A}}{{\partial z}}\right) , \end{aligned}$$

where $\tau$ represents time, *z* denotes the position along the nerve axon, and $\Delta \rho _A$ is the difference in nerve axon area density between the gel state $\rho _A$ and the fluid state $\rho _A^0.$ The sound velocity $c = \sqrt{\frac{1}{{\kappa ^{A}_{s} \rho ^{A}}}}$ depends on density and is determined by the properties of the nerve axon. Here, $\kappa ^A$ represents a constant related to the hydrodynamic Euler equation.

We add a frequency-dependent sound velocity that permits the generation of solitons to the Heimburg model in order to account for dispersion and nonlinear effects. To accomplished this, the sound speed equation was modified^1^ as follows:

2 $$\begin{aligned} c^2 = c_0^2 + \alpha \Delta \rho ^A + \beta \left( \Delta \rho ^A\right) ^2, \end{aligned}$$

where $c_0$ represents the velocity of small amplitude sound, $\alpha < 0$ and $\beta > 0$ are constants, and $\Delta \rho ^A$ is the difference in density between the gel and fluid states. We include a higher-order term $-h\frac{{\partial ^4 \Delta \rho ^A}}{{\partial z^4}}$ to Eq. (1) to account for mechanical dispersion. As a result, the following equation describes how sound travels through nerve axons,

3 $$\begin{aligned} \frac{{\partial ^2 \Delta \rho ^A}}{{\partial \tau ^2}} = \frac{{\partial }}{{\partial z}}\left( c_0^2 + \alpha \Delta \rho ^A + \beta (\Delta \rho ^A)^2\right) \frac{{\partial \Delta \rho ^A}}{{\partial z}} + u\frac{{\partial ^2 \Delta \rho ^A}}{{\partial z^2}}\frac{{\partial \Delta \rho ^A}}{{\partial \tau }} - h\frac{{\partial ^4 \Delta \rho ^A}}{{\partial z^4}}, \end{aligned}$$

where $$c_0 = \frac{1}{{K^A_s \rho ^A_0,}}~~~ \quad \alpha = -\frac{1}{{K^A_s (\rho ^A_0)^2}},~~~ \quad \text {and} \quad \beta = \frac{1}{{K^A_s (\rho ^A_0)^3}}$$.

Consider the dimensionless variables *u*, *x*, and *t* given as: $\upsilon = \frac{{\Delta \rho ^A}}{{\rho ^A_0}},~~~ \quad x = \frac{{c_0 z}}{{\sqrt{h}}},~~~ \quad t = \frac{{c_0^2 \tau }}{{\sqrt{h}}}$

Using these new variables, we arrive at the dimensionless density-wave equation from Eq. (3) as follows^1^:

4 $$\begin{aligned} \frac{{\partial ^2 \upsilon }}{{\partial t^2}} = \frac{{\partial }}{{\partial x}} \left( \left( 1 + b_{1} \upsilon + b_{2} \upsilon ^2\right) \frac{{\partial \upsilon }}{{\partial x}} \right) - \frac{{\partial ^4 \upsilon }}{{\partial x^4}} + m_{2} \frac{{\partial ^3 \upsilon }}{{\partial x^2 \partial t}}, \end{aligned}$$

with $m_{2} = \frac{u}{\sqrt{h}},~~~ \quad b_{2} =\frac{ \left( \rho ^A_0\right) ^2 }{ c_0^2}\beta ,~~~ and ~~~ b_{1} = \frac{\rho ^A_0}{ c_0^2}\alpha$. Equation (4) is our required Heimburg model that we used in this paper. In the next section, we give an overview about the Hirota bilinear method.

## An overview of the method

This section we outline the method used in the formation of the model for this paper. We start by first stating the general form of the nonlinear partial differential equation (NLPDE)

5 $$\begin{aligned} W(\upsilon , \upsilon _x, \upsilon _t, \upsilon _{xx}, \upsilon _{xt}, . . . )=0, \end{aligned}$$

in this case, *W* is a polynomial in $\upsilon (x, t)$. The following are the main phases in this method:

1. Consider the following transformation^32–35^:
  6 $$\begin{aligned} \upsilon (x,t)=\lambda (\eta ),~~~~~~\eta =x-m_{1}t, \end{aligned}$$
  where $m_{1}$ denotes the density pulse’s velocity. Equation (6) converts Equation (5) into the following form:
  7 $$\begin{aligned} U(\lambda ,\lambda ^{'},\lambda ^{''},\lambda ^{'''} ,. . .) = 0, \end{aligned}$$
  for polynomial *U* in $\lambda (\eta )$ having derivatives $\lambda ^{'}(\eta ),~\lambda ^{''}(\eta ),~\lambda ^{'''}(\eta )$, etc is twice integrable differential equation.
2. Assume Eq. (7) has a solution of the form:
  8 $$\begin{aligned} \lambda =\frac{ f^{\prime }(\eta )}{f(\eta )}, \end{aligned}$$
  where $f(\eta )$ is an unidentified function to be determined for our required solutions.
3. In this phase, we will determine $\lambda ^{'},~~\lambda ^{''},~~\lambda ^{'''},~.~.~.$, of Eq. (8) and substitute them into Eq. (7) to obtain an equation in terms of $f(\eta )$ and its derivatives upto the fifth order. We then integrate the resulting equation twice to to obtained our Bilinear form.
4. The numerous wave structures under consideration are now inserted into the Bilinear equation generated in phase (3). Then, in each case, we expand, simplify, and collect like terms and equate them to 0. Finally, in each situation, we solve the set of equations to obtain the suitable solutions.

## Application of the method on the Heimburg model

In this section, we used the previously stated stages to determine the various soliton generation and propagation in biomembranes and nerves for the Heimburg model.

We begin by considering Eq. (4), the dimensionless density-wave model. We now transform Eq. (4) to an ODE using the transformation $\upsilon (x,t)=\lambda (\eta )$, with $\eta =x-m_{1}t$, where $m_{1}$ is the velocity of the density pulse to obtain:

9 $$\begin{aligned} m_{1}\lambda ^{\prime \prime }-b_{1}(\lambda ^{\prime })^{2}-b_{2}\lambda (\lambda ^{\prime })^{2}-\lambda ^{\prime \prime }-b_{1}\lambda \lambda ^{\prime \prime }-b_{2}\lambda ^{2} \lambda ^{\prime \prime }+\lambda ^{\prime \prime \prime \prime }+m_{1}m_{2}\lambda ^{\prime \prime \prime }=0. \end{aligned}$$

Next we transform Eq. (9) from an ODE to a bilinear form using the the transformation

10 $$\begin{aligned} \lambda =\frac{ f^{\prime }(\eta )}{f(\eta )}, \end{aligned}$$

Substituting Eq. (10) into the Eq. (9) and integrating twice we obtained the bilinear form such as

11 $$\begin{aligned} -2 \left( b_2-6\right) f'^3-3 f f' \left( b_1 f'+6 f''+2 m_1 m_2 f'\right) +6 f^2 \left( f^{'''}+m_1 m_2 f''+\left( m_1^2-1\right) f'\right) =0. \end{aligned}$$

We now substitute the different wave structures we are studying into Eq. (11). Then, in each case, we expand, simplify, and collect like terms and equate them to 0. Finally, in each situation, we solve the set of equations to obtain the suitable solutions.

1. Homoclinic breather: we find some solutions using the homoclinic breather transformation such ass^10,36^:

12 $$\begin{aligned} f=\exp \left( -v \left( d_1 \eta +d_2\right) \right) +w_1 \exp \left( v \left( d_3 \eta +d_4\right) \right) +w_2 \cos \left( v \left( d_5 \eta +d_6\right) \right) . \end{aligned}$$

Substituting Eq. (12) and its derivatives to the third order into Eq. (9), simplifying and combining like terms using exponential, trigonometric, and exponential-trigonometric functions, and setting each of the resulting expressions to 0, we can determine the values of some of the parameters as follows:

Family 1:   the constant values are taken as $d_1=-d_3,~~~d_5=-\frac{d_3 \sqrt{3 m_2^2 m_1^2+4 m_1^2-4}}{m_1 m_2},~~~v=\frac{m_1 m_2}{2 d_3},~~~b_1=-\frac{2 \left( m_2^2 m_1^2-3 m_1^2+3\right) }{m_1 m_2},~~~b_2=\frac{6 \left( m_2^2 m_1^2-m_1^2+1\right) }{m_1^2 m_2^2}$, while $w_1,~~ w_2, ~~d_2, ~~ d_4, ~~ d_6$ are the free parameters.

Substituting them in Eq. (12) and then in Eq. (10) the result is, obtained such as

13 $$\begin{aligned} \lambda _{1,1}(\eta ) =\frac{\frac{1}{2} m_1 m_2 e^{-\frac{m_1 m_2 \left( d_2-d_3 \eta \right) }{2 d_3}}+\frac{1}{2} m_1 m_2 w_1 e^{\frac{m_1 m_2 \left( d_3 \eta +d_4\right) }{2 d_3}}+\frac{1}{2} \sqrt{3 m_2^2 m_1^2+4 m_1^2-4} w_2 \sin \left( G\right) }{e^{-\frac{m_1 m_2 \left( d_2-d_3 \eta \right) }{2 d_3}}+w_1 e^{\frac{m_1 m_2 \left( d_3 \eta +d_4\right) }{2 d_3}}+w_2 \cos \left( G\right) }, \end{aligned}$$

where $G=\frac{m_1 m_2 \left( d_6-\frac{d_3 \eta \sqrt{3 m_2^2 m_1^2+4 m_1^2-4}}{m_1 m_2}\right) }{2 d_3}.$ The breather wave solution to Eq. (4) is gained as:

14 $$\begin{aligned} \upsilon _{1,1}(x,t)=\frac{\sqrt{m_1^2 \left( 3 m_2^2+4\right) -4} w_2 e^{\frac{m_1 m_2 \left( d_3 \left( m_1 t-x\right) +d_2\right) }{2 d_3}} \sin \left( K\right) +m_1 m_2 \left( w_1 e^{\frac{\left( d_2+d_4\right) m_1 m_2}{2 d_3}}+1\right) }{2 \left( w_2 e^{\frac{m_1 m_2 \left( d_3 \left( m_1 t-x\right) +d_2\right) }{2 d_3}} \cos \left( K\right) +w_1 e^{\frac{\left( d_2+d_4\right) m_1 m_2}{2 d_3}}+1\right) }, \end{aligned}$$

where $$K=\frac{d_6 m_1 m_2}{2 d_3}+\frac{1}{2} \sqrt{m_1^2 \left( 3 m_2^2+4\right) -4} \left( m_1 t-x\right) .$$

Family 2:  the constant values are taken as $w_1=0,~~~d_1=\frac{\sqrt{m_1^2 \left( 9 m_2^2+8\right) -8}-3 m_1 m_2}{4 v},~~~d_5=\frac{\sqrt{\frac{m_1^2 m_2^2 \left( -\left( 9 m_2^2+4\right) m_1^2+3 \sqrt{m_1^2 \left( 9 m_2^2+8\right) -8} m_2 m_1+4\right) }{v^2}}}{2 \sqrt{2} m_1 m_2},~~~b_1=-8 m_1 m_2,~~~b_2=-\frac{3 \left( \left( 3 m_2^2-4\right) m_1^2+\sqrt{m_1^2 \left( 9 m_2^2+8\right) -8} m_2 m_1+4\right) }{2 \left( m_1^2-1\right) }$ while $w_2, ~~d_2, ~~d_3, ~~ d_4, ~~ d_6$ are the free parameters.

Substituting them in Eq. (12) and then in Eq. (10) the result is, obtained such as

15 $$\begin{aligned} \lambda _{1,2}(\eta ) =\frac{\frac{1}{4} \left( 3 m_1 m_2-\sqrt{m_1^2 \left( 9 m_2^2+8\right) -8}\right) e^{ -v \left( d_2+\frac{\eta \left( \sqrt{m_1^2 \left( 9 m_2^2+8\right) -8}-3 m_1 m_2\right) }{4 v}\right) }- J \sin \left( G\right) }{e^{-v \left( d_2+\frac{\eta \left( \sqrt{m_1^2 \left( 9 m_2^2+8\right) -8}-3 m_1 m_2\right) }{4 v}\right) }+w_2 \cos \left( G\right) }. \end{aligned}$$

The breather wave solution to Eq. (4) is gained as:

16 $$\begin{aligned} \upsilon _{1,2}(x,t)=\frac{\frac{1}{4} \left( 3 m_1 m_2-\sqrt{m_1^2 \left( 9 m_2^2+8\right) -8}\right) e^{\frac{1}{4} \left( \sqrt{m_1^2 \left( 9 m_2^2+8\right) -8}-3 m_1 m_2\right) \left( m_1 t-x\right) -d_2 v}-J \sin \left( G\left( x-m_1 t\right) \right) }{e^{\frac{1}{4} \left( \sqrt{m_1^2 \left( 9 m_2^2+8\right) -8}-3 m_1 m_2\right) \left( m_1 t-x\right) -d_2 v}+w_2 \cos \left( G\left( x-m_1 t\right) \right) }, \end{aligned}$$

where $$G=v \left( d_6+\frac{\eta \sqrt{\frac{m_1^2 m_2^2 \left( -\left( 9 m_2^2+4\right) m_1^2+3 \sqrt{m_1^2 \left( 9 m_2^2+8\right) -8} m_2 m_1+4\right) }{v^2}}}{2 \sqrt{2} m_1 m_2}\right)$$

and $$J=\frac{v w_2 \sqrt{\frac{m_1^2 m_2^2 \left( -\left( 9 m_2^2+4\right) m_1^2+3 \sqrt{m_1^2 \left( 9 m_2^2+8\right) -8} m_2 m_1+4\right) }{v^2}}}{2 \sqrt{2} m_1 m_2}.$$

Family 3:   the constant values are taken as $d_1=-d_3,~~~d_5= -i d_3,~~~v=-\frac{\sqrt{m_1^2 \left( 9 m_2^2+8\right) -8}-3 m_1 m_2}{4 d_3},~~~b_1=-8 m_1 m_2$ while $w_1,~~ w_2, ~~d_2, ~~ d_4, ~~ d_6,~~ b_2$ are the free parameters.

Substituting them in Eq. (12) and then in Eq. (10) the result is, obtained such as

17 $$\begin{aligned} \lambda _{1,3}(\eta ) =\frac{\frac{G}{d_3} e^{G\left( d_2-d_3 \eta \right) }+\frac{G}{d_3} G w_1 e^{-G \left( d_3 \eta +d_4\right) }+\frac{G}{d_3} i G w_2 \sin \left( G \left( d_6-i d_3 \eta \right) \right) }{e^{ G \left( d_2-d_3 \eta \right) }+w_1 e^ {-G \left( d_3 \eta +d_4\right) }+w_2 \cos \left( G \left( d_6-i d_3 \eta \right) \right) }. \end{aligned}$$

The breather wave solution to Eq. (4) is gained as:

18 $$\begin{aligned} \upsilon _{1,3}(x,t)=-\frac{\frac{G}{d_3} \left( -i w_2 e^ {\left( -G\left( d_3 \left( x-m_1 t\right) +d_4\right) \right) } \sin \left( G \left( d_6-i d_3 \left( x-m_1 t\right) \right) \right) +e^{\left( d_2+d_4\right) G}+w_1\right) }{4 \left( w_2 e^{ -G \left( d_3 \left( x-m_1 t\right) +d_4\right) }\cos \left( G \left( d_6-i d_3 \left( x-m_1 t\right) \right) \right) +e^ {G\left( d_2+d_4\right) }+w_1\right) }, \end{aligned}$$

where $$G=\frac{\left( 3 m_1 m_2-\sqrt{m_1^2 \left( 9 m_2^2+8\right) -8}\right) }{4 d_3}.$$

2. Interaction via double exponents: we find double exponents solutions by using the transformation such as^10,36^:

19 $$\begin{aligned} f=w_1 \exp \left( d_1 \eta +d_2\right) +w_2 \exp \left( d_3 \eta +d_4\right) . \end{aligned}$$

By substituting Eq. (19) and its derivatives to the third order into Eq. (9), simplifying and collecting like terms exponential functions with the same powers, and setting each of the resulting expressions to 0, we can determine the values of some of the parameters as follows:

Family 1:   the constant values are taken as $d_1=\frac{1}{2} \left( -\sqrt{m_2^2 m_1^2-4 m_1^2+4}-m_1 m_2\right) ,~~~d_3=0,~~~b_1=3 \sqrt{m_2^2 m_1^2-4 m_1^2+4}+m_1 m_2,~~~b_2=6,$ while $w_1,~~ w_2, ~~d_2, ~~ d_4,$ are the free parameters.

Substituting them in Eq. (19) and then in Eq. (10) the result is, obtained such as

20 $$\begin{aligned} \lambda _{2,1}(\eta ) =\frac{\left( -\sqrt{m_2^2 m_1^2-4 m_1^2+4}-m_1 m_2\right) w_1 \exp \left( d_2+\frac{1}{2} \eta \left( -\sqrt{m_2^2 m_1^2-4 m_1^2+4}-m_1 m_2\right) \right) }{2 \left( w_1 \exp \left( d_2+\frac{1}{2} \eta \left( -\sqrt{m_2^2 m_1^2-4 m_1^2+4}-m_1 m_2\right) \right) +e^{d_4} w_2\right) }. \end{aligned}$$

The interaction of double exponents solution to Eq. (4) is gained as:

21 $$\begin{aligned} \upsilon _{2,1}(x,t)=-\frac{\left( \sqrt{\left( m_2^2-4\right) m_1^2+4}+m_1 m_2\right) w_1 \exp \left( d_2+\frac{1}{2} \left( \sqrt{\left( m_2^2-4\right) m_1^2+4}+m_1 m_2\right) \left( m_1^2 t-x\right) \right) }{2 \left( w_1 \exp \left( d_2-\frac{1}{2} \left( \sqrt{\left( m_2^2-4\right) m_1^2+4}+m_1 m_2\right) \left( x-m_1^2 t\right) \right) +e^{d_4} w_2\right) }. \end{aligned}$$

Family 2:   the constant values are taken as $d_1=\frac{1}{2} \left( -\sqrt{\left( m_1 m_2-d_3\right) {}^2-4 \left( 1-m_1^2\right) }+d_3-m_1 m_2\right) ,~~~b_1=\frac{-d_3 \sqrt{\left( m_1 m_2-d_3\right) {}^2-4 \left( 1-m_1^2\right) }+d_3 m_1 m_2-d_3^2+2 m_1^2-2}{d_3},~~~b_2=\frac{3 \left( d_3 \sqrt{\left( m_1 m_2-d_3\right) {}^2-4 \left( 1-m_1^2\right) }-d_3 m_1 m_2+d_3^2\right) }{2 d_3^2}$, while $w_1,~~ w_2, ~~d_2, ~~d_3, ~~ d_4,$ are the free parameters.

Substituting them in Eq. (19) and then in Eq. (10) the result is, obtained such as

22 $$\begin{aligned} \lambda _{2,2}(\eta ) =\frac{\frac{1}{2} w_1 \left( -\sqrt{\left( m_1 m_2-d_3\right) {}^2-4 \left( 1-m_1^2\right) }+d_3-m_1 m_2\right) e^{\frac{1}{2} \eta \left( -\sqrt{\left( m_1 m_2-d_3\right) {}^2-4 \left( 1-m_1^2\right) }+d_3-m_1 m_2\right) +d_2}+d_3 w_2 e^{d_3 \eta +d_4}}{w_1 e^ {\frac{1}{2} \eta \left( -\sqrt{\left( m_1 m_2-d_3\right) {}^2-4 \left( 1-m_1^2\right) }+d_3-m_1 m_2\right) +d_2}+w_2 e^{d_3 \eta +d_4}}. \end{aligned}$$

The interaction of double exponents solution to Eq. (4) is gained as:

23 $$\begin{aligned} \upsilon _{2,2}(x,t)=\frac{d_3 w_2 e^{d_3 \left( x-m_1^2 t\right) +d_4}-\frac{1}{2} w_1 \left( \sqrt{\left( d_3-m_1 m_2\right) {}^2+4 \left( m_1^2-1\right) }-d_3+m_1 m_2\right) e^ {d_2-\frac{1}{2} \left( \sqrt{\left( d_3-m_1 m_2\right) {}^2+4 \left( m_1^2-1\right) }-d_3+m_1 m_2\right) \left( x-m_1^2 t\right) }}{w_1 e^{ d_2-\frac{1}{2} \left( \sqrt{\left( d_3-m_1 m_2\right) {}^2+4 \left( m_1^2-1\right) }-d_3+m_1 m_2\right) \left( x-m_1^2 t\right) }+w_2 e^{d_3 \left( x-m_1^2 t\right) +d_4}}. \end{aligned}$$

3. Lump periodic: we find some solutions using the function^36^

24 $$\begin{aligned} f=\left( d_1 \eta +d_2\right) {}^2+\left( d_3 \eta +d_4\right) {}^2+\cos \left( d_5 \eta +d_6\right) +d_7. \end{aligned}$$

By substituting Eq. (24) and its derivatives to the third order into Eq. (9), simplifying and collecting like terms $\eta$, exponential functions, and trigonometric functions with the same powers, and setting each of the resulting expressions to 0, we can determine the values of some of the parameters as follows:

Family 1:  the constant values are taken as $d_1=0,~~~d_3=0,~~~d_5=-\frac{\sqrt{1-m_1^2}}{\sqrt{2}},~~~d_7=-d_2^2-d_4^2,~~~b_1=-2 m_1 m_2,~~~b_2=6$, while $d_2,~~d_4,$ are free parameters.

Substituting them in Eq. (24) and then in Eq. (10) the result is, obtained such as

25 $$\begin{aligned} \lambda _{3,1}(\eta ) =\frac{\sqrt{1-m_1^2}}{\sqrt{2}} \tan \left( d_6-\frac{\eta \sqrt{1-m_1^2}}{\sqrt{2}}\right) . \end{aligned}$$

The Lump wave solution to Eq. (4) is gained as:

26 $$\begin{aligned} \upsilon _{3,1}(x,t)=\frac{\sqrt{1-m_1^2}}{\sqrt{2}} \tan \left( d_6+\frac{\sqrt{1-m_1^2} \left( m_1 t-x\right) }{\sqrt{2}}\right) . \end{aligned}$$

Family 2:   the constant values are taken as $d_3=-\frac{d_1 d_2}{d_4},~~~d_5=0,~~~d_7=-d_2^2-d_4^2$, while $d_2,~~d_4,$ are free parameters.

Substituting them in Eq. (24) and then in Eq. (10) the result is, obtained such as

27 $$\begin{aligned} \lambda _{3,2}(\eta ) =\frac{2 d_1 \left( d_1 \eta +d_2\right) -\frac{2 d_1 d_2 \left( d_4-\frac{d_1 d_2 \eta }{d_4}\right) }{d_4}}{\left( d_1 \eta +d_2\right) {}^2+\left( d_4-\frac{d_1 d_2 \eta }{d_4}\right) {}^2-d_2^2-d_4^2+\cos \left( d_6\right) }. \end{aligned}$$

The Lump wave solution to Eq. (4) is gained as:

28 $$\begin{aligned} \upsilon _{3,2}(x,t)=\frac{2 d_1^2 \left( d_2^2+d_4^2\right) \left( x-m_1 t\right) }{d_1^2 \left( d_2^2+d_4^2\right) \left( x-m_1 t\right) {}^2+d_4^2 \cos \left( d_6\right) }. \end{aligned}$$

4. Mixed type: we find mixed type solutions by using the transformation such as^10,36^:

29 $$\begin{aligned} f=w_1 \exp \left( v \left( d_1 \eta +d_2\right) \right) +w_2 \exp \left( -v \left( d_1 \eta +d_2\right) \right) +w_3 \sin \left( v \left( d_3 \eta +d_4\right) \right) +w_4 \sinh \left( v \left( d_5 \eta +d_6\right) \right) . \end{aligned}$$

By substituting Eq. (29) and its derivatives to the third order into Eq. (10), simplifying and collecting like terms, exponential functions, trigonometric functions and hyperbolic functions with the same powers, and setting each of the resulting expressions to 0, we can determine the values of some of the parameters as follows:

Family 1: the constant values are taken as $w_1=0,~~~w_2=0,~~~d_3=\frac{\sqrt{1-m_1^2}}{\sqrt{2} v},~~~d_5=\frac{\sqrt{m_1^2-1}}{\sqrt{2} v},~~~b_1=-2 m_1 m_2,~~~b_2=6,$ while $d_4,~~d_6, ~~ w_3, ~~w_4, ~~v,$ are free parameters.

Substituting them in Eq. (29) and then in Eq. (10) the result is, obtained such as

30 $$\begin{aligned} \lambda _{4,1}(\eta ) =\frac{\frac{\sqrt{1-m_1^2} w_3 \cos \left( v \left( d_4+\frac{\eta \sqrt{1-m_1^2}}{\sqrt{2} v}\right) \right) }{\sqrt{2}}+\frac{\sqrt{m_1^2-1} w_4 \cosh \left( v \left( d_6+\frac{\eta \sqrt{m_1^2-1}}{\sqrt{2} v}\right) \right) }{\sqrt{2}}}{w_3 \sin \left( v \left( d_4+\frac{\eta \sqrt{1-m_1^2}}{\sqrt{2} v}\right) \right) +w_4 \sinh \left( v \left( d_6+\frac{\eta \sqrt{m_1^2-1}}{\sqrt{2} v}\right) \right) }. \end{aligned}$$

The mixed type solution to Eq. (4) is gained as:

31 $$\begin{aligned} \upsilon _{4,1}(x,t)=\frac{\sqrt{1-m_1^2} w_3 \cos \left( d_4 v+\frac{1}{2} \sqrt{2-2 m_1^2} \left( x-m_1 t\right) \right) +\sqrt{m_1^2-1} w_4 \cosh \left( d_6 v+\frac{\sqrt{m_1^2-1} \left( x-m_1 t\right) }{\sqrt{2}}\right) }{\sqrt{2} \left( w_3 \sin \left( d_4 v+\frac{1}{2} \sqrt{2-2 m_1^2} \left( x-m_1 t\right) \right) +w_4 \sinh \left( d_6 v+\frac{\sqrt{m_1^2-1} \left( x-m_1 t\right) }{\sqrt{2}}\right) \right) }. \end{aligned}$$

Family 2: The constant values are taken as $w_1=0,~~~d_1=\frac{\sqrt{m_1^2-1}}{\sqrt{2} v},~~~d_3=\frac{\sqrt{1-m_1^2}}{\sqrt{2} v},~~~d_5=\frac{\sqrt{m_1^2-1}}{\sqrt{2} v},~~~b_1=-2 m_1 m_2,~~~b_2=6$, while $d_2,~~d_4, ~~ d_6, ~~w_2,~~ w_3, ~~w_4,~~v,$ are free parameters.

Substituting them in Eq. (29) and then in Eq. (10) the result is, obtained such as

32 $$\begin{aligned} \lambda _{4,2}(\eta ) =\frac{-\frac{\sqrt{m_1^2-1} w_2 e^{-v \left( d_2+\frac{\eta \sqrt{m_1^2-1}}{\sqrt{2} v}\right) }}{\sqrt{2}}+\frac{\sqrt{1-m_1^2} w_3 \cos \left( v \left( d_4+\frac{\eta \sqrt{1-m_1^2}}{\sqrt{2} v}\right) \right) }{\sqrt{2}}+\frac{\sqrt{m_1^2-1} w_4 \cosh \left( v \left( d_6+\frac{\eta \sqrt{m_1^2-1}}{\sqrt{2} v}\right) \right) }{\sqrt{2}}}{w_2 e^{-v \left( d_2+\frac{\eta \sqrt{m_1^2-1}}{\sqrt{2} v}\right) }+w_3 \sin \left( v \left( d_4+\frac{\eta \sqrt{1-m_1^2}}{\sqrt{2} v}\right) \right) +w_4 \sinh \left( v \left( d_6+\frac{\eta \sqrt{m_1^2-1}}{\sqrt{2} v}\right) \right) }. \end{aligned}$$

The mixed type solution to Eq. (4) is gained as:

33 $$\begin{aligned} \upsilon _{4,2}(x,t)=\frac{e^{d_2 v+\frac{\sqrt{m_1^2-1} \left( x-m_1 t\right) }{\sqrt{2}}} \left( \sqrt{1-m_1^2} w_3 \cos \left( G\right) +\sqrt{m_1^2-1} w_4 \cosh \left( G\right) \right) -\sqrt{m_1^2-1} w_2}{\sqrt{2} \left( e^{d_2 v+\frac{\sqrt{m_1^2-1} \left( x-m_1 t\right) }{\sqrt{2}}} \left( w_3 \sin \left( d_4 v+\frac{1}{2} \sqrt{2-2 m_1^2} \left( x-m_1 t\right) \right) +w_4 \sinh \left( G\right) \right) +w_2\right) }. \end{aligned}$$

Where $$G=d_6 v+\frac{\sqrt{m_1^2-1} \left( x-m_1 t\right) }{\sqrt{2}}.$$

Family 3: The constant values are taken as $w_1=0,~~~w_4=0,~~~d_3=-\frac{\sqrt{1-m_1^2}}{\sqrt{2} v},~~~b_1=-2 m_1 m_2,~~~b_2=\frac{3 \left( 2 d_1^2 v^2+m_1^2-1\right) }{2 d_1^2 v^2}$, while $d_1,~~d_4, ~~d_5,~~ d_6, ~~w_2,~~ w_3, ~~v,$ are free parameters.

Substituting them in Eq. (29) and then in Eq. (10) the result is, obtained such as

34 $$\begin{aligned} \lambda _{4,3}(\eta ) =\frac{d_1 v w_2 \left( -e^{-v \left( d_1 \eta +d_2\right) }\right) -\frac{\sqrt{1-m_1^2} w_3 \cos \left( v \left( d_4-\frac{\eta \sqrt{1-m_1^2}}{\sqrt{2} v}\right) \right) }{\sqrt{2}}}{w_3 \sin \left( v \left( d_4-\frac{\eta \sqrt{1-m_1^2}}{\sqrt{2} v}\right) \right) +w_2 e^{-v \left( d_1 \eta +d_2\right) }}. \end{aligned}$$

The mixed type solution to Eq. (4) is gained as:

35 $$\begin{aligned} \upsilon _{4,3}(x,t)=-\frac{\sqrt{2-2 m_1^2} w_3 e^{v \left( d_1 \left( x-m_1 t\right) +d_2\right) } \cos \left( d_4 v+\frac{1}{2} \sqrt{2-2 m_1^2} \left( m_1 t-x\right) \right) +2 d_1 v w_2}{2 \left( w_3 e^{v \left( d_1 \left( x-m_1 t\right) +d_2\right) } \sin \left( d_4 v+\frac{1}{2} \sqrt{2-2 m_1^2} \left( m_1 t-x\right) \right) +w_2\right) }. \end{aligned}$$

Family 4: the constant values are taken as $w_2=0,~~~w_4=0,~~~v=\frac{\sqrt{1-m_1^2}}{\sqrt{2} d_3},~~~b_1= -2 m_1 m_2$, while $d_1,~~d_2, ~~d_4,~~ ~~w_2,~~ w_3, ~~v,$ are free parameters.

Substituting them in Eq. (29) and then in Eq. (10) the result is, obtained such as

36 $$\begin{aligned} \lambda _{4,4}(\eta ) =\frac{\frac{d_1 \sqrt{1-m_1^2} w_1 e^{\frac{\sqrt{1-m_1^2} \left( d_1 \eta +d_2\right) }{\sqrt{2} d_3}}}{\sqrt{2} d_3}+\frac{\sqrt{1-m_1^2} w_3 \cos \left( \frac{\sqrt{1-m_1^2} \left( d_3 \eta +d_4\right) }{\sqrt{2} d_3}\right) }{\sqrt{2}}}{w_1 e^{\frac{\sqrt{1-m_1^2} \left( d_1 \eta +d_2\right) }{\sqrt{2} d_3}}+w_3 \sin \left( \frac{\sqrt{1-m_1^2} \left( d_3 \eta +d_4\right) }{\sqrt{2} d_3}\right) }. \end{aligned}$$

The mixed type solution to Eq. (4) is gained as:

37 $$\begin{aligned} \upsilon _{4,4}(x,t)=\frac{\sqrt{1-m_1^2} \left( d_1 w_1 \exp \left( \frac{\sqrt{1-m_1^2} \left( d_1 \left( x-m_1 t\right) +d_2\right) }{\sqrt{2} d_3}\right) +d_3 w_3 \cos \left( \frac{\sqrt{1-m_1^2} \left( d_3 \left( x-m_1 t\right) +d_4\right) }{\sqrt{2} d_3}\right) \right) }{\sqrt{2} d_3 \left( w_1 \exp \left( \frac{\sqrt{1-m_1^2} \left( d_1 \left( x-m_1 t\right) +d_2\right) }{\sqrt{2} d_3}\right) +w_3 \sin \left( \frac{\sqrt{1-m_1^2} \left( d_3 \left( x-m_1 t\right) +d_4\right) }{\sqrt{2} d_3}\right) \right) }. \end{aligned}$$

5. Multiwave: we find multiwave solutions by using the transformation such as^10,36^:

38 $$\begin{aligned} f=w_2 \cos \left( d_3 \eta +d_4\right) +w_1 \cosh \left( d_1 \eta +d_2\right) +w_3 \cosh \left( d_5 \eta +d_6\right) . \end{aligned}$$

By substituting Eq. (38) and its derivatives to the third order into Eq. (9), simplifying and collecting like terms of trigonometric functions and hyperbolic functions with the same powers, and setting each of the resulting expressions to 0, we can determine the values of some of the parameters as follows:

Family 1: the constant values are taken as $w_3=0,~~~d_1=\frac{\sqrt{m_1^2-1}}{\sqrt{2}},~~~d_3=\frac{\sqrt{1-m_1^2}}{\sqrt{2}}$, while $d_2, ~~d_4,~~ w_1, ~~w_2,$ are free parameters.

Substituting them in Eq. (38) and then in Eq. (10) the result is, obtained such as

39 $$\begin{aligned} \lambda _{5,1}(\eta ) =\frac{\frac{\sqrt{m_1^2-1} w_1 \sinh \left( d_2+\frac{\eta \sqrt{m_1^2-1}}{\sqrt{2}}\right) }{\sqrt{2}}-\frac{\sqrt{1-m_1^2} w_2 \sin \left( d_4+\frac{\eta \sqrt{1-m_1^2}}{\sqrt{2}}\right) }{\sqrt{2}}}{w_2 \cos \left( d_4+\frac{\eta \sqrt{1-m_1^2}}{\sqrt{2}}\right) +w_1 \cosh \left( d_2+\frac{\eta \sqrt{m_1^2-1}}{\sqrt{2}}\right) }. \end{aligned}$$

The multiwave solution to Eq. (4) is gained as:

40 $$\begin{aligned} \upsilon _{5,1}(x,t)=\frac{\sqrt{m_1^2-1} w_1 \sinh \left( d_2+\frac{\sqrt{m_1^2-1} \left( x-m_1 t\right) }{\sqrt{2}}\right) -\sqrt{1-m_1^2} w_2 \sin \left( d_4+\frac{\sqrt{1-m_1^2} \left( x-m_1 t\right) }{\sqrt{2}}\right) }{\sqrt{2} \left( w_2 \cos \left( d_4+\frac{\sqrt{1-m_1^2} \left( x-m_1 t\right) }{\sqrt{2}}\right) +w_1 \cosh \left( d_2+\frac{\sqrt{m_1^2-1} \left( x-m_1 t\right) }{\sqrt{2}}\right) \right) }. \end{aligned}$$

Family 2: the constant values are taken as $w_3=0, ~~d_1=0,~~~d_3=-\frac{\sqrt{1-m_1^2}}{\sqrt{2}}$, while $d_2, ~~d_4,~~ w_1, ~~w_2,$ are free parameters.

Substituting them in Eq. (38) and then in Eq. (10) the result is, obtained such as

41 $$\begin{aligned} \lambda _{5,2}(\eta ) =\frac{\sqrt{1-m_1^2} w_2 \sin \left( d_4-\frac{\eta \sqrt{1-m_1^2}}{\sqrt{2}}\right) }{\sqrt{2} \left( w_2 \cos \left( d_4-\frac{\eta \sqrt{1-m_1^2}}{\sqrt{2}}\right) +w_1 \cosh \left( d_2\right) \right) }. \end{aligned}$$

The multiwave solution to Eq. (4) is gained as:

42 $$\begin{aligned} \upsilon _{5,2}(x,t)=\frac{\sqrt{1-m_1^2} w_2 \sin \left( d_4-\frac{\sqrt{1-m_1^2} \left( x-m_1 t\right) }{\sqrt{2}}\right) }{\sqrt{2} \left( w_2 \cos \left( d_4-\frac{\sqrt{1-m_1^2} \left( x-m_1 t\right) }{\sqrt{2}}\right) +w_1 \cosh \left( d_2\right) \right) }. \end{aligned}$$

Family 3: the constant values are taken as $w_2=0,~~~w_3=0,~~~d_1=\frac{\sqrt{m_1^2-1}}{\sqrt{2}}$, while $d_2, ~~d_4,~~ w_1,$ are free parameters.

Substituting them in Eq. (38) and then in Eq. (10) the result is, obtained such as

43 $$\begin{aligned} \lambda _{5,3}(\eta ) =\frac{\sqrt{m_1^2-1}}{\sqrt{2}} \tanh \left( d_2+\frac{\eta \sqrt{m_1^2-1}}{\sqrt{2}}\right) . \end{aligned}$$

The multiwave solution to Eq. (4) is gained as:

44 $$\begin{aligned} \upsilon _{5,3}(x,t)=\frac{\sqrt{m_1^2-1}}{\sqrt{2}} \tanh \left( d_2+\frac{\sqrt{m_1^2-1} \left( x-m_1 t\right) }{\sqrt{2}}\right) . \end{aligned}$$

Family 4: the constant values are taken as $w_1=0,~~~w_3=0,~~~d_3=-\frac{\sqrt{1-m_1^2}}{\sqrt{2}}$, while $d_4,~~ w_2,$ are free parameters.

Substituting them in Eq. (38) and then in Eq. (10) the result is, obtained such as

45 $$\begin{aligned} \lambda _{5,4}(\eta ) =\frac{\sqrt{1-m_1^2}}{\sqrt{2}} \tan \left( d_4-\frac{\eta \sqrt{1-m_1^2}}{\sqrt{2}}\right) . \end{aligned}$$

The multiwave solution to Eq. (4) is gained as:

46 $$\begin{aligned} \upsilon _{5,4}(x,t)=\frac{\sqrt{1-m_1^2}}{\sqrt{2}} \tan \left( d_4-\frac{\sqrt{1-m_1^2} \left( x-m_1 t\right) }{\sqrt{2}}\right) . \end{aligned}$$

6. Periodic cross kink: we find periodic cross kink solutions by using the transformation such as^10,36^:

47 $$\begin{aligned} f=\exp \left( -v \left( d_1 \eta +d_2\right) \right) +w_1 \exp \left( v \left( d_3 \eta +d_4\right) \right) +w_2 \cos \left( v \left( d_5 \eta +d_6\right) \right) +w_3 \cosh \left( v \left( d_7 \eta +d_8\right) \right) +d_9. \end{aligned}$$

By substituting Eq. (47) and its derivatives to the third order into Eq. (9), simplifying and collecting like terms of exponential functions, trigonometric functions and hyperbolic functions with the same powers, and setting each of the resulting expressions to 0, we can determine the values of some of the parameters as follows:

Family 1: the constant values are taken as $w_2=0,~~~d_1=-d_3,~~~d_7=\frac{\sqrt{\frac{1}{3} d_3 v \left( \left( b_2-9\right) d_3 v+6 m_1 m_2\right) +m_1^2-1}}{v},~~~d_9=0,~~~b_1=-\frac{2 \left( b_2 d_3^2 v^2-3 m_1^2+3\right) }{3 d_3 v}$, while $d_2,~~d_4,~~d_6,~~ w_1,~~w_3, ~~v$ are free parameters.

Substituting them in Eq. (47) and then in Eq. (10) the result is, obtained such as

48 $$\begin{aligned} \lambda _{6,1}(\eta ) =\frac{-\frac{v \left( G\right) }{4 \left( b_2 v^2+2 v^2\right) } e^{-v \left( \frac{\eta \left( G\right) }{4 \left( b_2 v^2+2 v^2\right) }+d_2\right) }-\frac{1}{2} \sqrt{m_1^2-1} w_2 \sin \left( v \left( d_6+\frac{\eta \sqrt{m_1^2-1}}{2 v}\right) \right) }{e^ {-v \left( \frac{\eta \left( G\right) }{4 \left( b_2 v^2+2 v^2\right) }+d_2\right) }+w_2 \cos \left( v \left( d_6+\frac{\eta \sqrt{m_1^2-1}}{2 v}\right) \right) +w_3 \cosh \left( d_8 v\right) }. \end{aligned}$$

where $$G=\sqrt{\left( -4 b_1 v-9 m_1 m_2 v\right) {}^2-4 \left( m_1^2-1\right) \left( 2 b_2 v^2+4 v^2\right) }+4 b_1 v+9 m_1 m_2 v.$$

The periodic cross kink solution to Eq. (4) is gained as:

49 $$\begin{aligned} \upsilon _{6,1}(x,t)=\frac{\sqrt{3} w_3 J e^{v \left( d_3 \left( m_1 t-x\right) +d_2\right) } \sinh \left( \left( x-m_1 t\right) J+d_8 v\right) +3 d_3 v \left( w_1 e^{\left( d_2+d_4\right) v}+1\right) }{3 \left( w_3 e^{v \left( d_3 \left( m_1 t-x\right) +d_2\right) } \cosh \left( \left( x-m_1 t\right) J+d_8 v\right) +w_1 e^{\left( d_2+d_4\right) v}+1\right) }, \end{aligned}$$

where $$J=\sqrt{\left( b_2-9\right) d_3^2 v^2+6 d_3 m_1 m_2 v+3 \left( m_1^2-1\right) }.$$

Family 2: the constant values are taken as $w_1=0,~~~w_2=0,~~~d_7=-\frac{\sqrt{3 d_1^2 v^2-2 m_1^2+2}}{\sqrt{2} v},~~~b_1=-2 m_1 m_2,~~~b_2=6$, while $d_1,~~d_2,~~d_8,~~d_9,~~w_3, ~~v$ are free parameters.

Substituting them in Eq. (47) and then in Eq. (10) the result is, obtained such as

50 $$\begin{aligned} \lambda _{6,2}(\eta ) =\frac{d_1 v \left( -e^{-v \left( d_1 \eta +d_2\right) }\right) -\frac{w_3 \sqrt{3 d_1^2 v^2-2 m_1^2+2} \sinh \left( v \left( d_8-\frac{\eta \sqrt{3 d_1^2 v^2-2 m_1^2+2}}{\sqrt{2} v}\right) \right) }{\sqrt{2}}}{w_3 \cosh \left( v \left( d_8-\frac{\eta \sqrt{3 d_1^2 v^2-2 m_1^2+2}}{\sqrt{2} v}\right) \right) +e^{-v \left( d_1 \eta +d_2\right) }+d_9}. \end{aligned}$$

The periodic cross kink solution to Eq. (4) is gained as:

51 $$\begin{aligned} \upsilon _{6,2}(x,t)=-\frac{w_3 \sqrt{6 d_1^2 v^2-4 m_1^2+4} e^{v \left( d_1 \left( x-m_1 t\right) +d_2\right) } \sinh \left( \sqrt{\frac{3}{2} d_1^2 v^2-m_1^2+1} \left( m_1 t-x\right) +d_8 v\right) +2 d_1 v}{2 \left( w_3 e^{v \left( d_1 \left( x-m_1 t\right) +d_2\right) } \cosh \left( \sqrt{\frac{3}{2} d_1^2 v^2-m_1^2+1} \left( m_1 t-x\right) +d_8 v\right) +d_9 e^{v \left( d_1 \left( x-m_1 t\right) +d_2\right) }+1\right) }. \end{aligned}$$

Family 3: the constant values are taken as $w_1=0,~~~d_5=\frac{\sqrt{1-m_1^2}}{\sqrt{2} v},~~~d_7=\frac{\sqrt{1-m_1^2}}{2 v},~~~d_9=0,~~~b_1=-2 m_1 m_2,~~~b_2=6$, while $d_1,~~d_2,~~d_6,~~d_8,~~w_2,~~w_3, ~~v$ are free parameters.

Substituting them in Eq. (47) and then in Eq. (10) the result is, obtained such as

52 $$\begin{aligned} \lambda _{6,3}(\eta ) =\frac{-\frac{\sqrt{1-m_1^2} w_2 \sin \left( v \left( d_6+\frac{\eta \sqrt{1-m_1^2}}{\sqrt{2} v}\right) \right) }{\sqrt{2}}+\frac{1}{2} \sqrt{1-m_1^2} w_3 \sinh \left( v \left( d_8+\frac{\eta \sqrt{1-m_1^2}}{2 v}\right) \right) +d_1 v \left( -e^{-v \left( d_1 \eta +d_2\right) }\right) }{w_2 \cos \left( v \left( d_6+\frac{\eta \sqrt{1-m_1^2}}{\sqrt{2} v}\right) \right) +w_3 \cosh \left( v \left( d_8+\frac{\eta \sqrt{1-m_1^2}}{2 v}\right) \right) +e^{-v \left( d_1 \eta +d_2\right) }}. \end{aligned}$$

The periodic cross kink solution to Eq. (4) is gained as:

53 $$\begin{aligned} \upsilon _{6,3}(x,t)=\frac{\sqrt{1-m_1^2} e^{v \left( d_1 \left( x-m_1 t\right) +d_2\right) } \left( w_3 \sinh \left( d_8 v+\frac{1}{2} \sqrt{1-m_1^2} \left( x-m_1 t\right) \right) -\sqrt{2} w_2 \sin \left( d_6 v+\frac{1}{2} \sqrt{2-2 m_1^2} \left( x-m_1 t\right) \right) \right) -2 d_1 v}{2 \left( w_2 e^{v \left( d_1 \left( x-m_1 t\right) +d_2\right) } \cos \left( d_6 v+\frac{1}{2} \sqrt{2-2 m_1^2} \left( x-m_1 t\right) \right) +w_3 e^{v \left( d_1 \left( x-m_1 t\right) +d_2\right) } \cosh \left( d_8 v+\frac{1}{2} \sqrt{1-m_1^2} \left( x-m_1 t\right) \right) +1\right) }. \end{aligned}$$

## Graphical presentations

**Figure 1:**
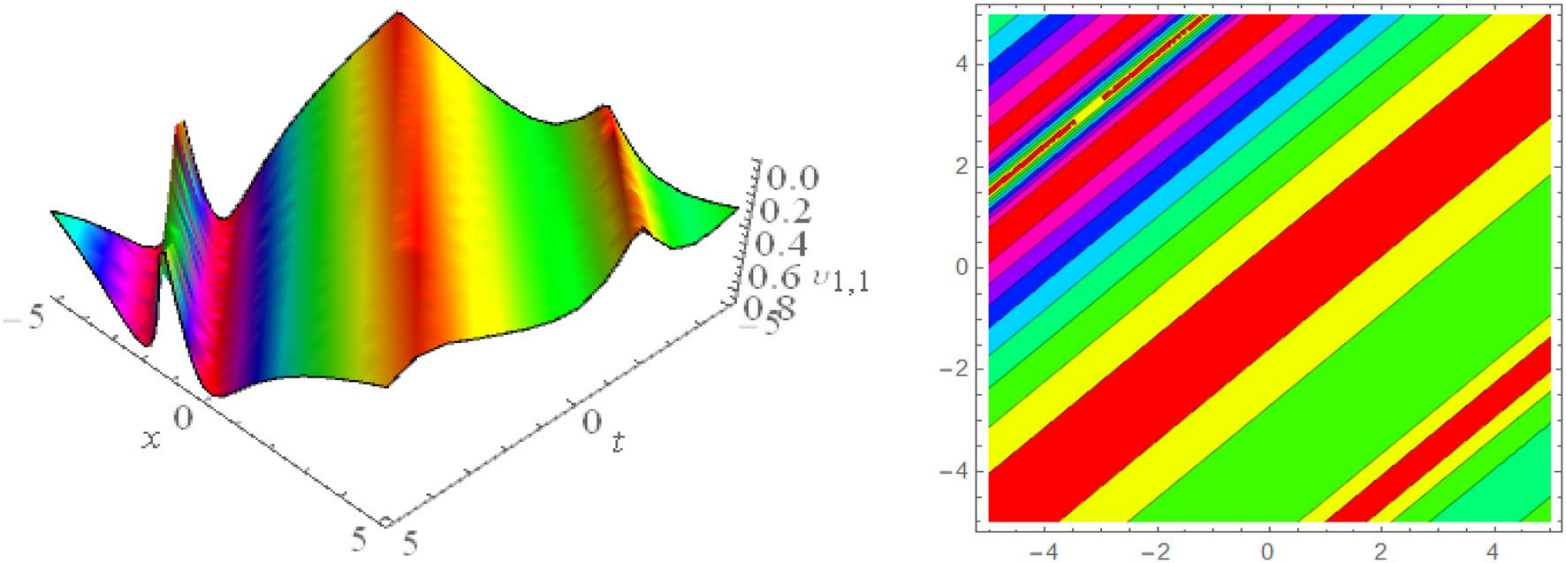
3D and corresponding contour for solution $\upsilon _{1,1}(x, t)$ with   $d_2=1.1,~~ d_3=0.5,~~ d_4=0.3,~~ d_6=2.3,~~m_1=1.1,~~m_2=0.06,~~ m_4=-1.9,~~ w_1=0.2,~~ w_2=0.8$.

**Figure 2:**
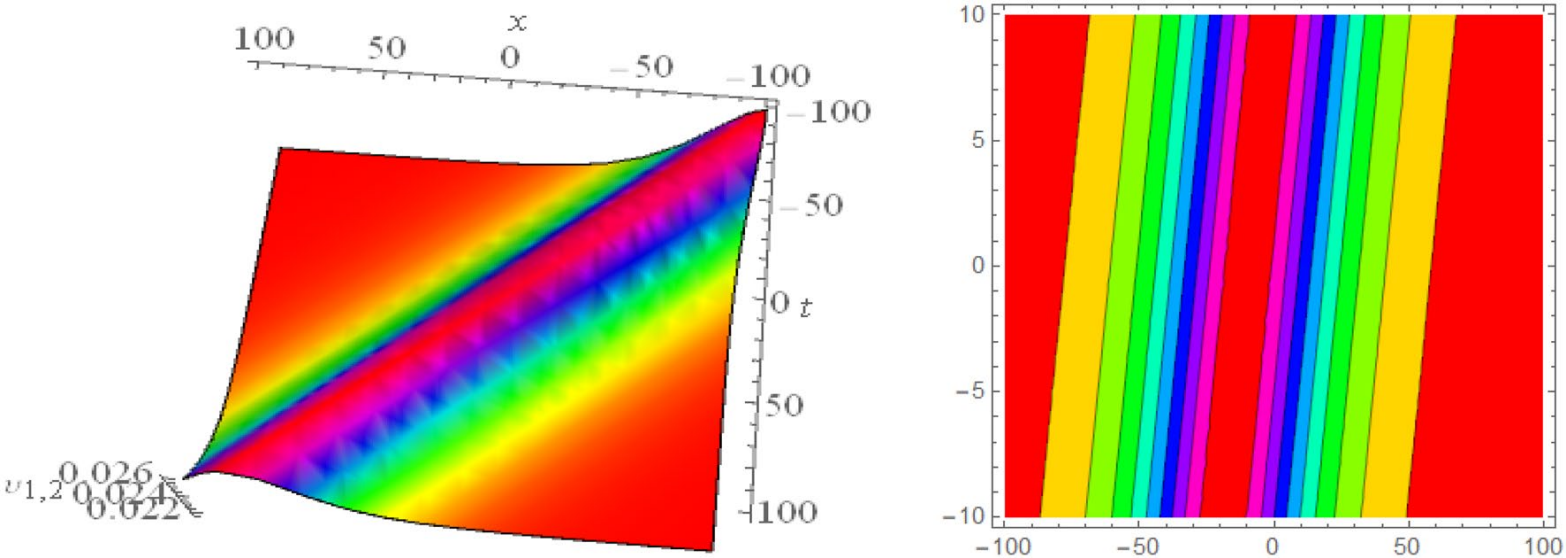
3D and corresponding contour for solution $\upsilon _{1,2}(x, t)$ with   $d_2=0.01,~~ d_3=1.5,~~d_4=2.3,~~ d_6=10.3,~~m_1=0.92,~~ m_2=2.6,~~m_4=1,~~ v=0.5,~~ w_1=0.2,~~w_2=2.5$.

**Figure 3:**
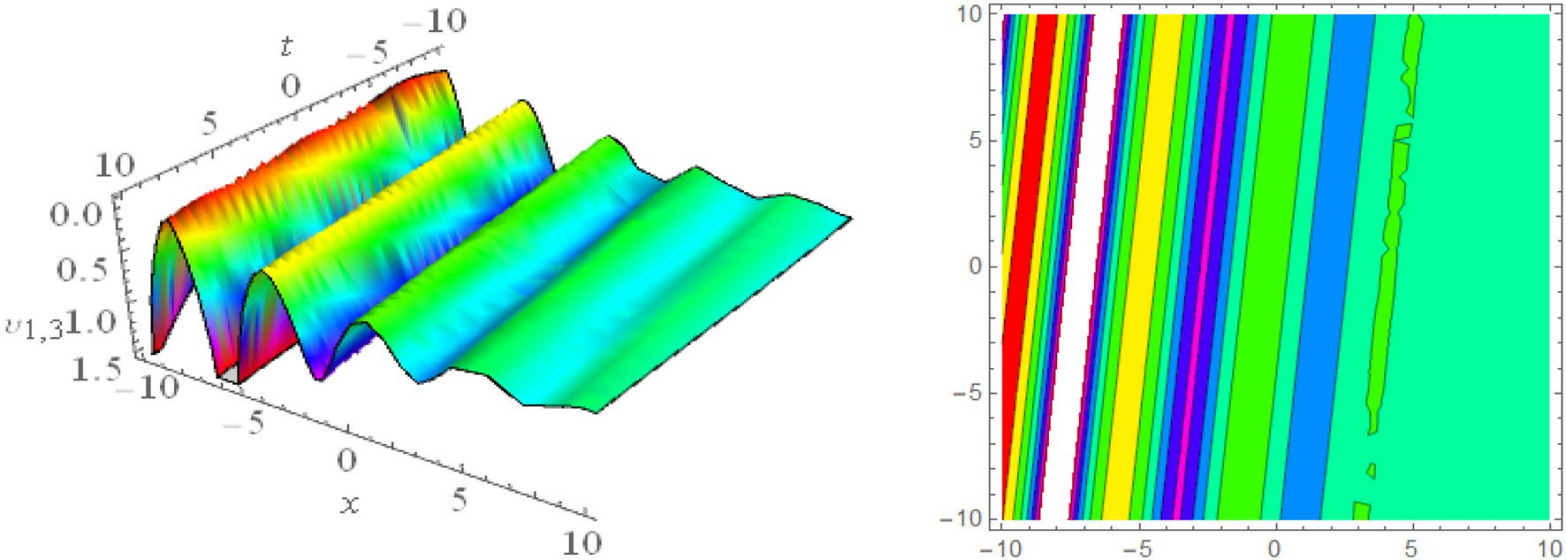
3D and corresponding contour for solution $\upsilon _{1,3}(x, t)$ with   $d_2=0.1,~~d_4=1,~~ d_3=1.01,~~d_6=0.3,~~ m_1=0.1,~~ m_2=1.1,~~m_4=1.6,~~ v=0.5,~~ w_1=10.2,~~w_2=6.5$.

**Figure 4:**
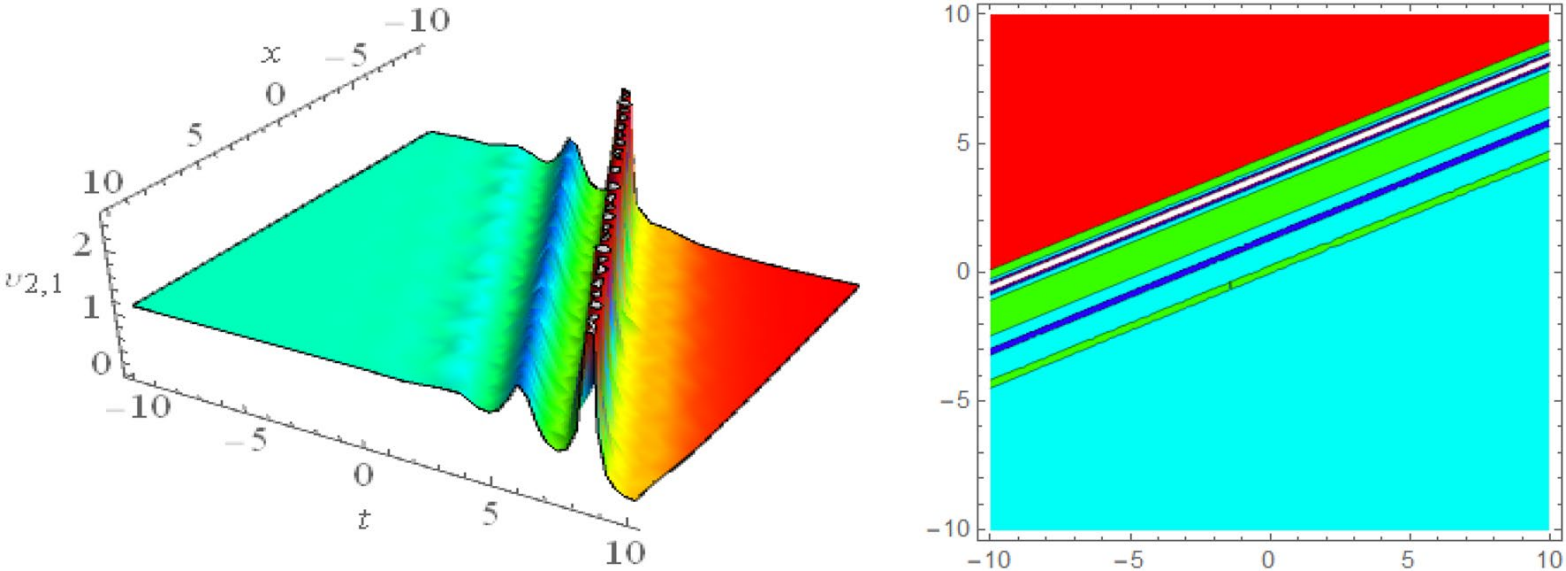
3D and corresponding contour for solution $\upsilon _{2,1}(x,t)$ with   $d_2=3.1,~~d_4=1.1,~~m_1=-1.5,~~m_2=0.36,~~w_1=3.5,~~w_2=3.2$.

**Figure 5:**
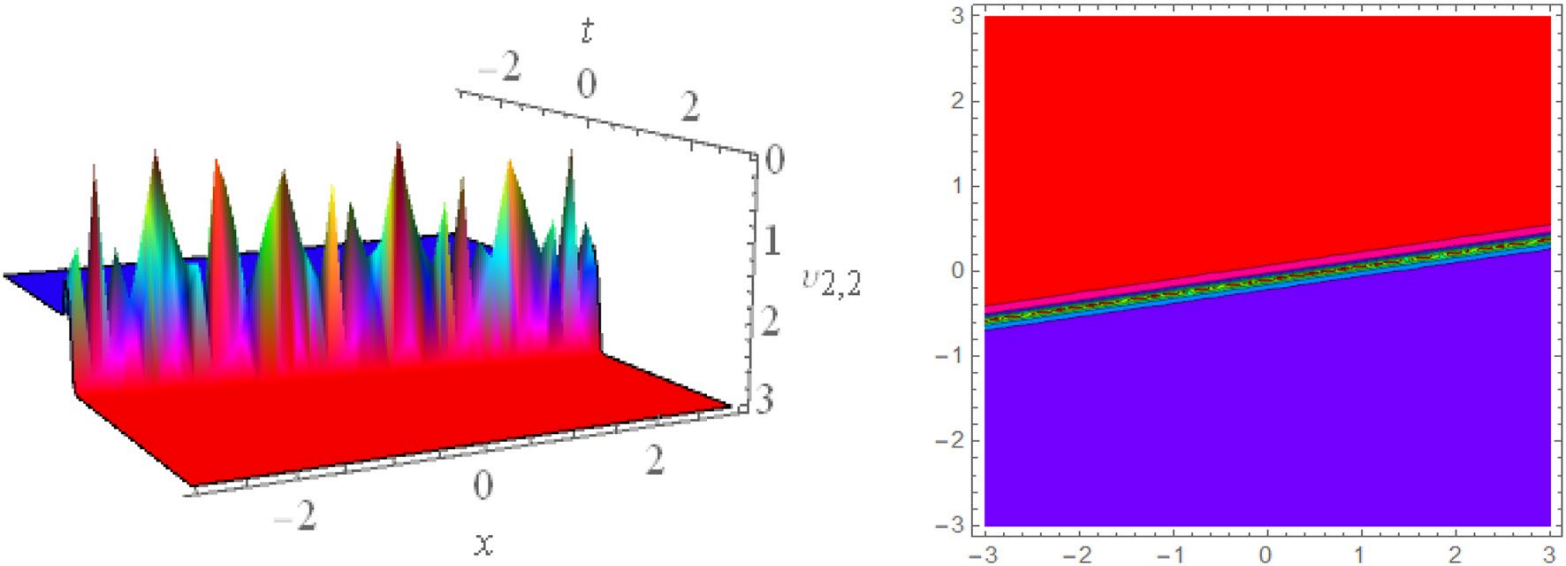
3D and corresponding contour for solution $\upsilon _{2,2}(x,t)$ with   $d_2=2.1,~~d_3=2.1,~~d_4=0.1,~~m_1=2.5,~~m_2=1.36,~~w_1=0.5,~~w_2=0.2$.

**Figure 6:**
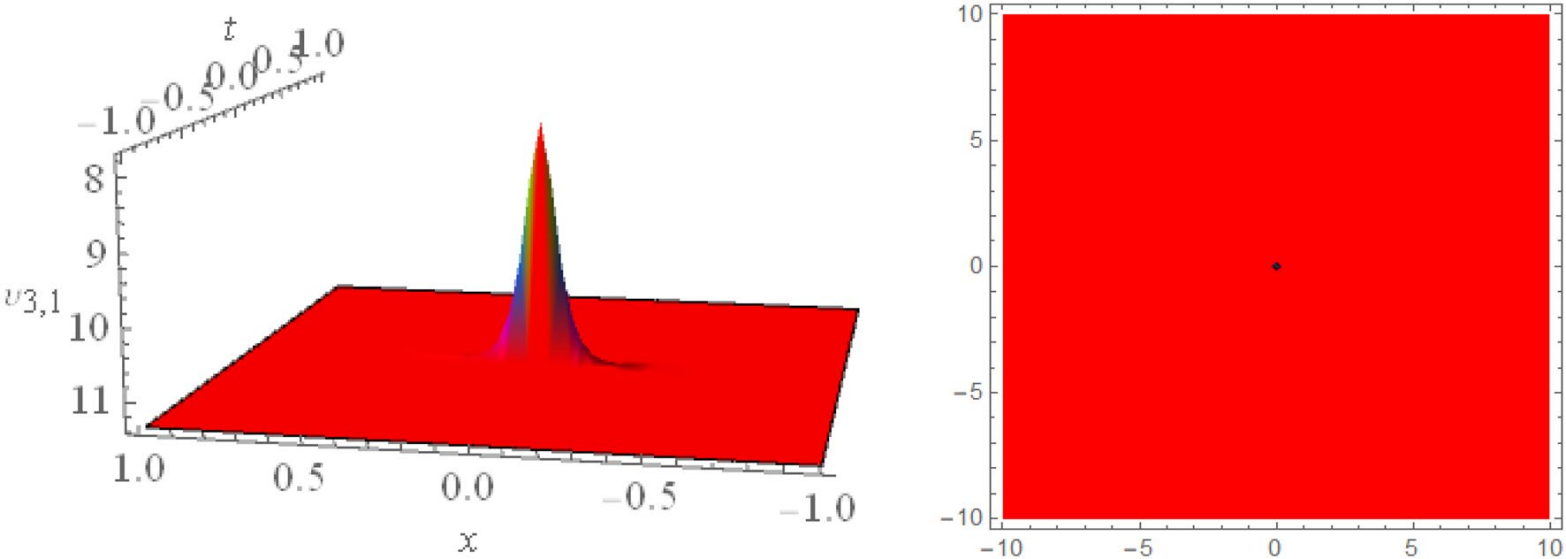
3D and corresponding contour for solution $\upsilon _{3,1}(x,t)$ with   $d_6=0.6,~~m_1=16.1$.

**Figure 7:**
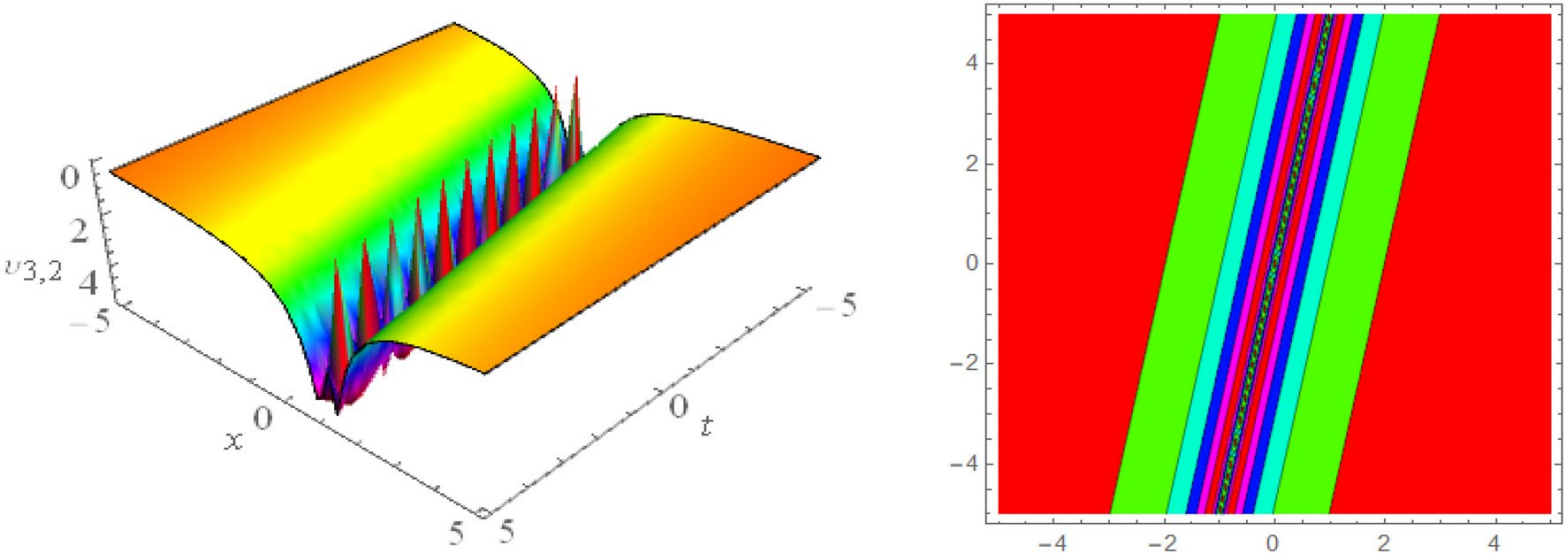
3D and corresponding contour for solution $\upsilon _{3,2}(x,t)$ with   $d_1=2.5,~~d_2=2.5,~~d_4=1.5,~~d_6=0.6,~~d_7=1.7,~~m_1=0.2$.

**Figure 8:**
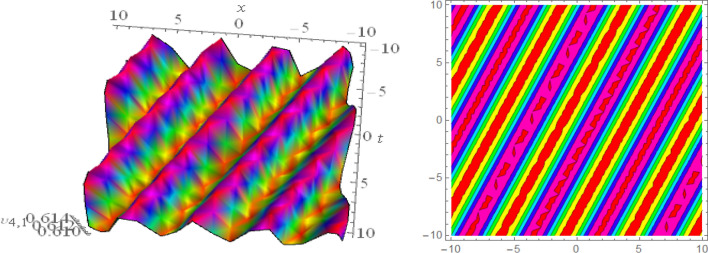
3D and corresponding contour for solution $\upsilon _{4,1}(x,t)$ with   $d_4=6.1,~~d_6=4.1,~~m_1=0.5,~~v=1.5,~~w_3=1.6,~~w_4=1.5$.

**Figure 9:**
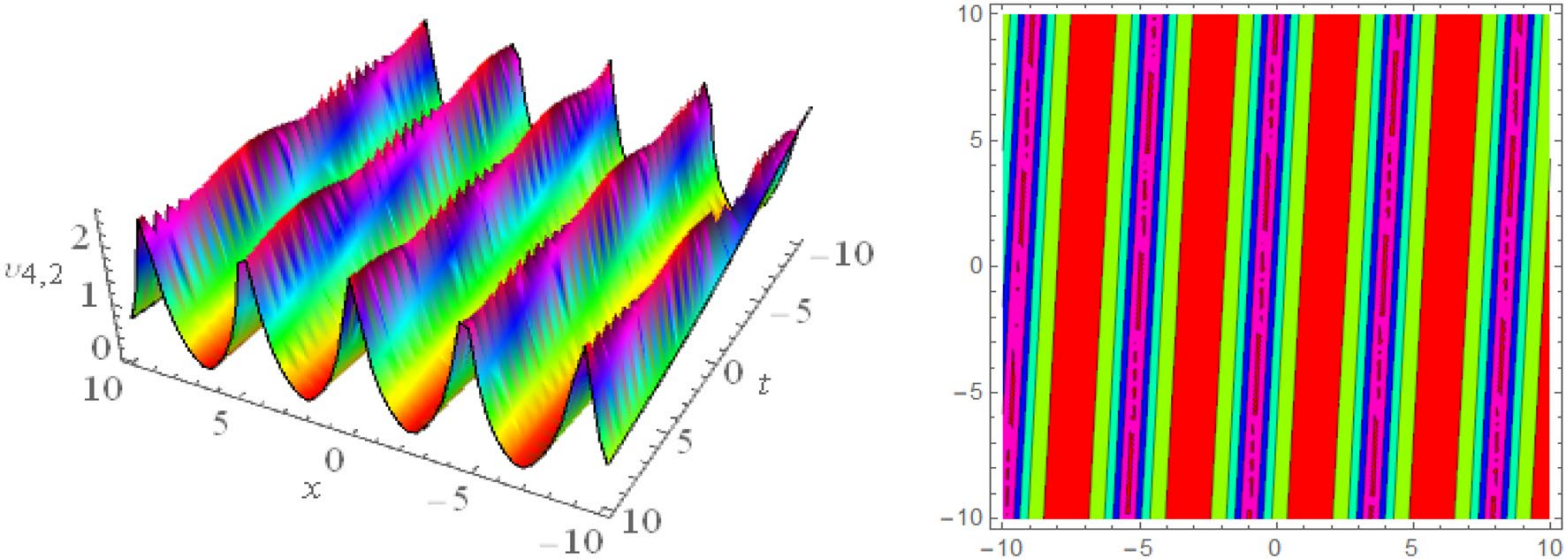
3D and corresponding contour for solution $\upsilon _{4,2}(x,t)$ with   $d_2=2.1,~~d_4=0.1,~~d_6=0.1,~~m_1=0.05,~~v=0.25,~~w_2=1.03,~~w_3=1.08,~~ w_4=1.6$.

**Figure 10:**
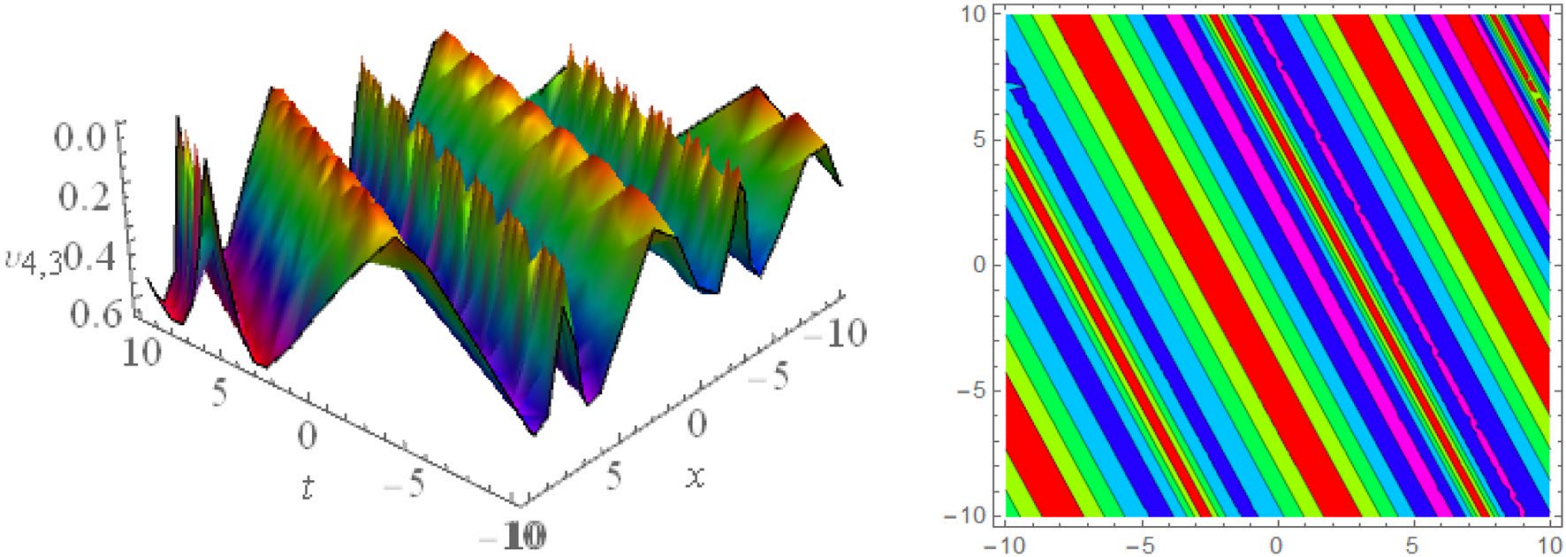
3D and corresponding contour for solution $\upsilon _{4,3}(x,t)$ with   $d_1=4.1,~~d_2=0.5,~~d_4=0.5,~~m_1=-0.5,~~v=0.003,~~w_2=1.6,~~w_3=0.99$.

**Figure 11:**
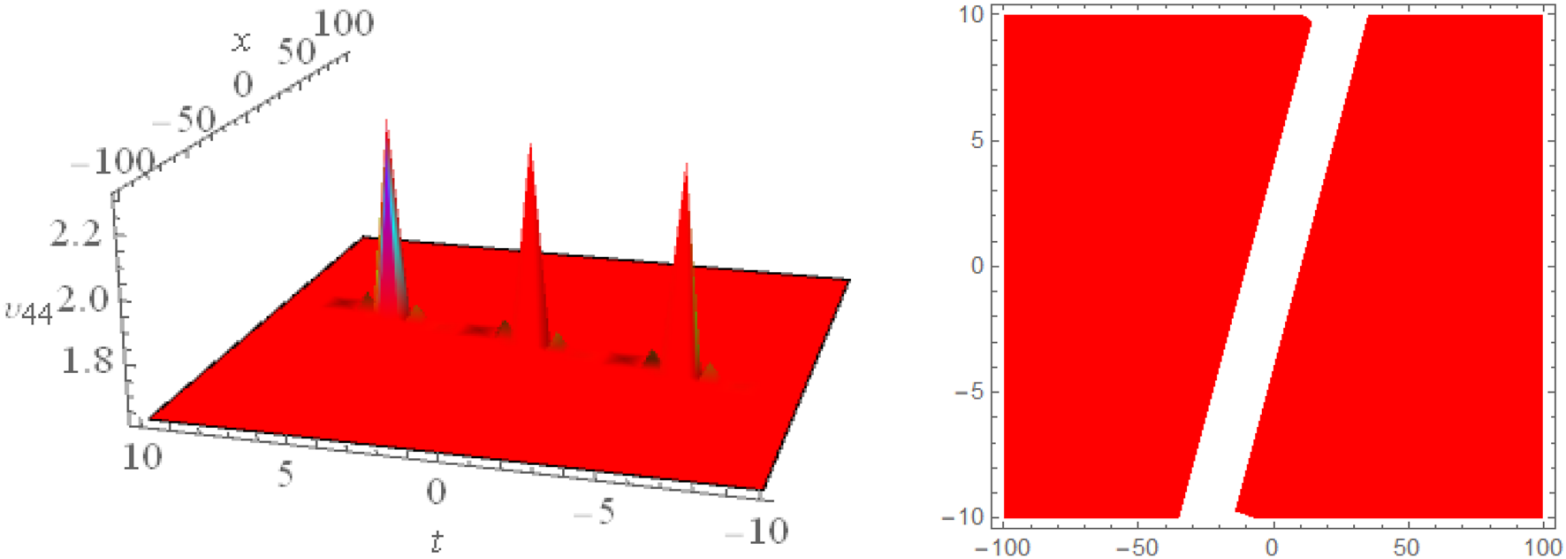
3D and corresponding contour for solution $\upsilon _{4,4}(x,t)$ with   $d_4=4.1,~~m_1=2.5$.

**Figure 12:**
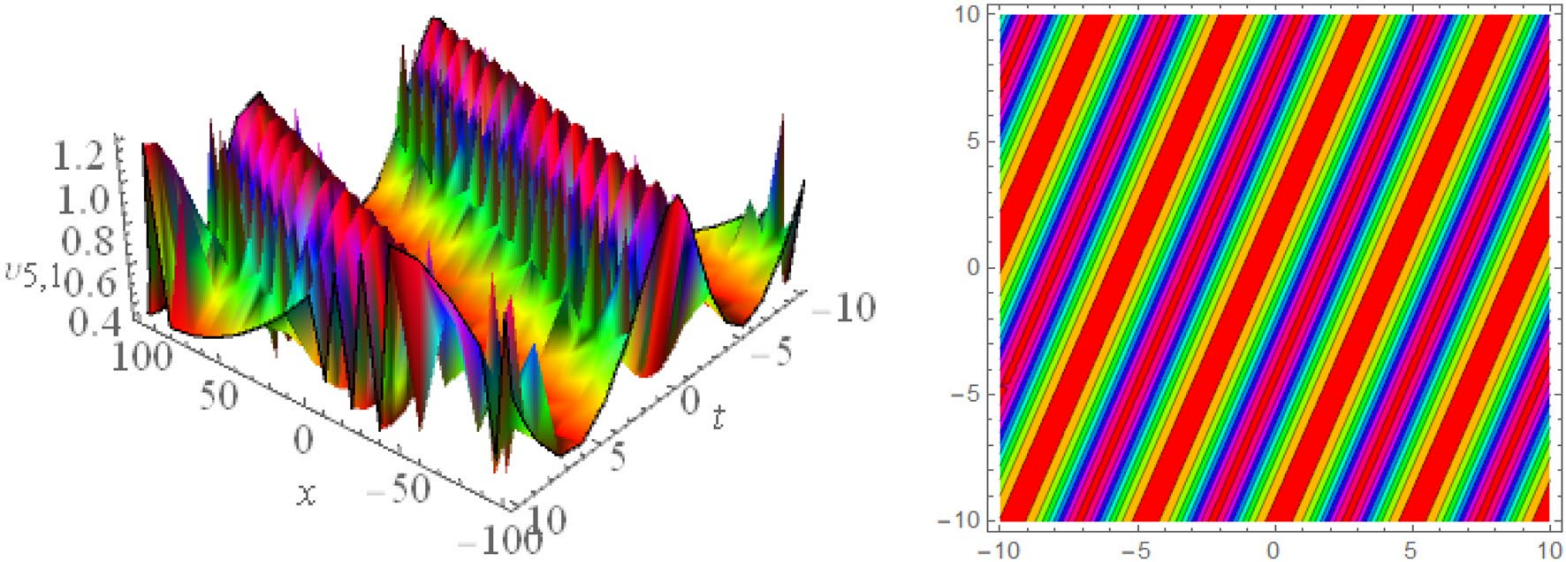
3D and corresponding contour for solution $\upsilon _{5,1}(x,t)$ with   $d_2=1.01,~~d_4=1.05,~~m_1=0.4,~~w_1=6.1,~~w_2=4.5$.

**Figure 13:**
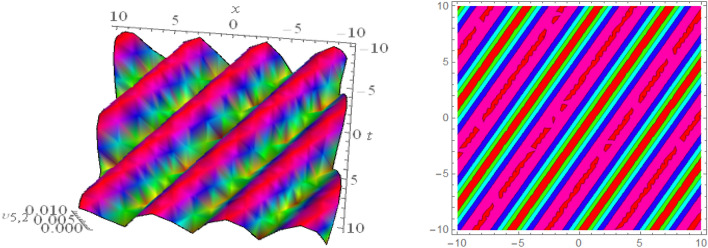
3D and corresponding contour for solution $\upsilon _{5,2}(x,t)$ with   $d_2=4.1,~~d_4=0.5,~~m_1=0.65,~~w_1=0.51,~~w_2=0.29$.

**Figure 14:**
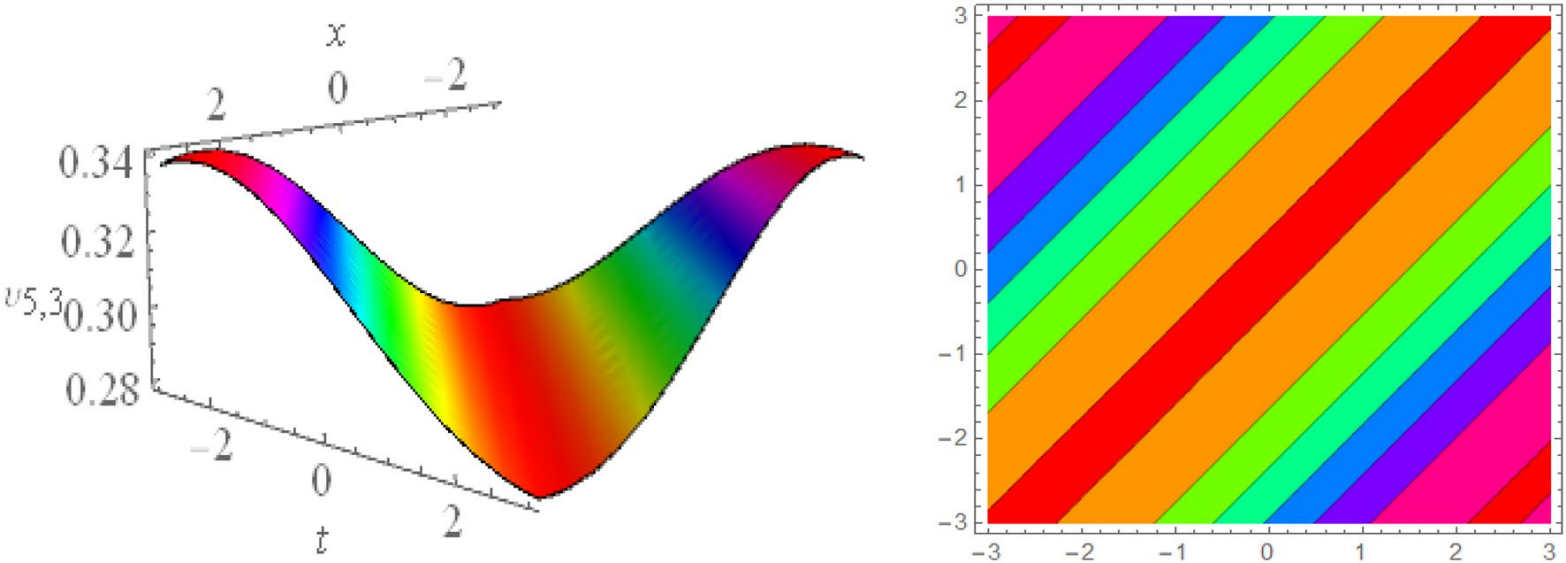
3D and corresponding contour for solution $\upsilon _{5,3}(x,t)$ with   $d_4=0.5,~~m_1=0.855$.

**Figure 15:**
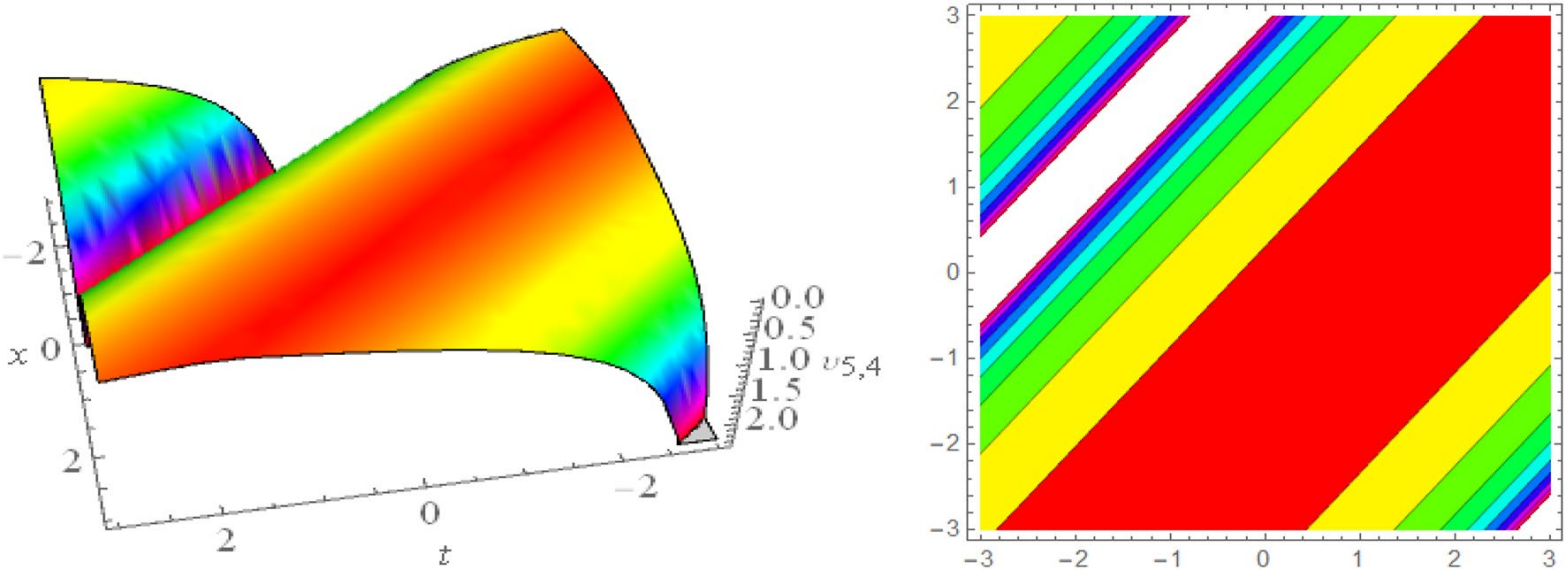
3D and corresponding contour for solution $\upsilon _{5,4}(x,t)$ with   $d_2=1.5,~~m_1=0.9$.

**Figure 16:**
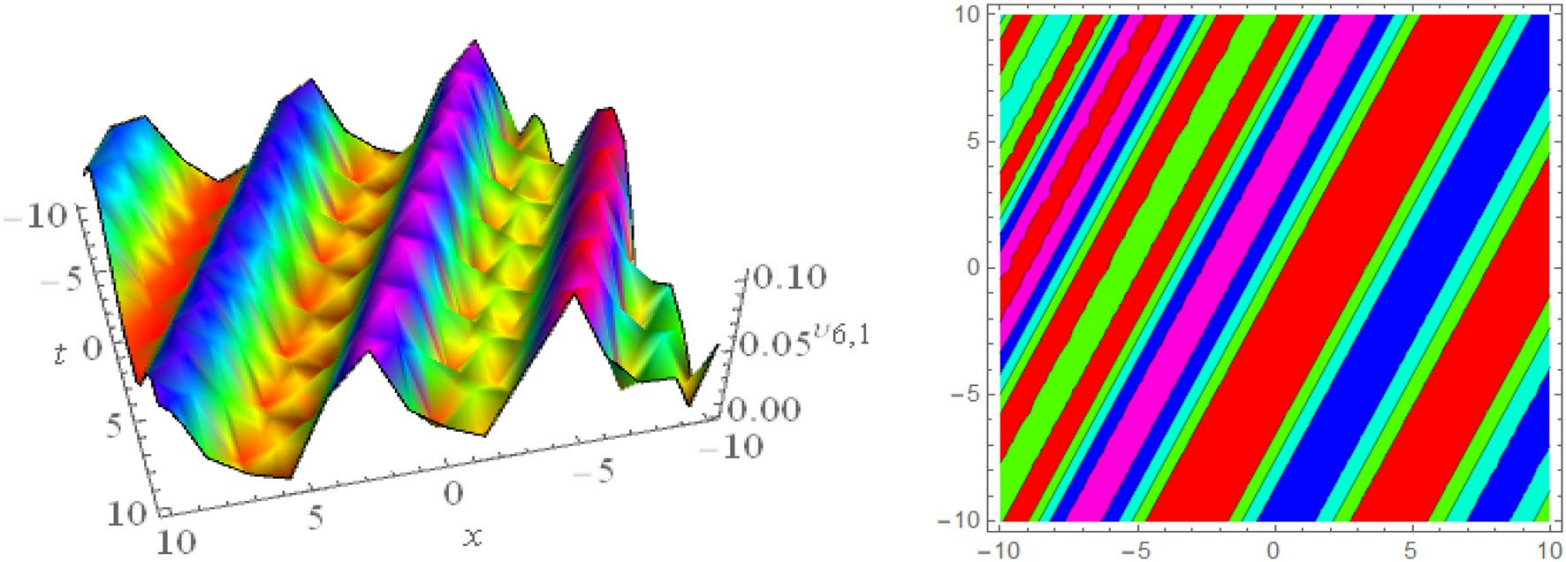
3D and corresponding contour for solution $\upsilon _{6,1}(x,t)$ with   $b_2=1.2,~~d_2=0.1,~~d_3=0.067,~~d_4=2.1,~~d_8=0.1,~~m_1=0.5,~~m_2=0.6,~~ v=0.5,~~w_3=0.4,~~w_1=1.98$.

**Figure 17:**
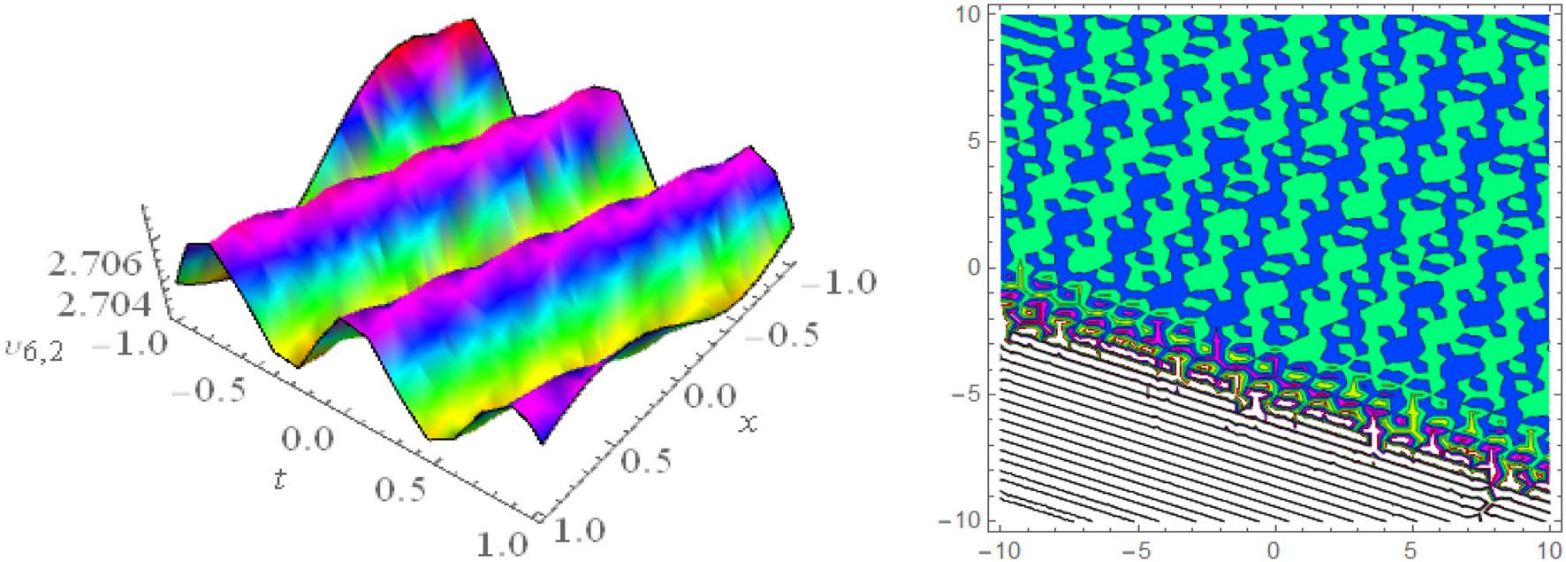
3D and corresponding contour for solution $\upsilon _{6,2}(x,t)$ with   $d_1=0.1,~~d_2=0.62,~~d_8=2.3,~~d_9=0.9,~~m_1=-2.9,~~v=2.5,~~w_3=8.6$.

**Figure 18:**
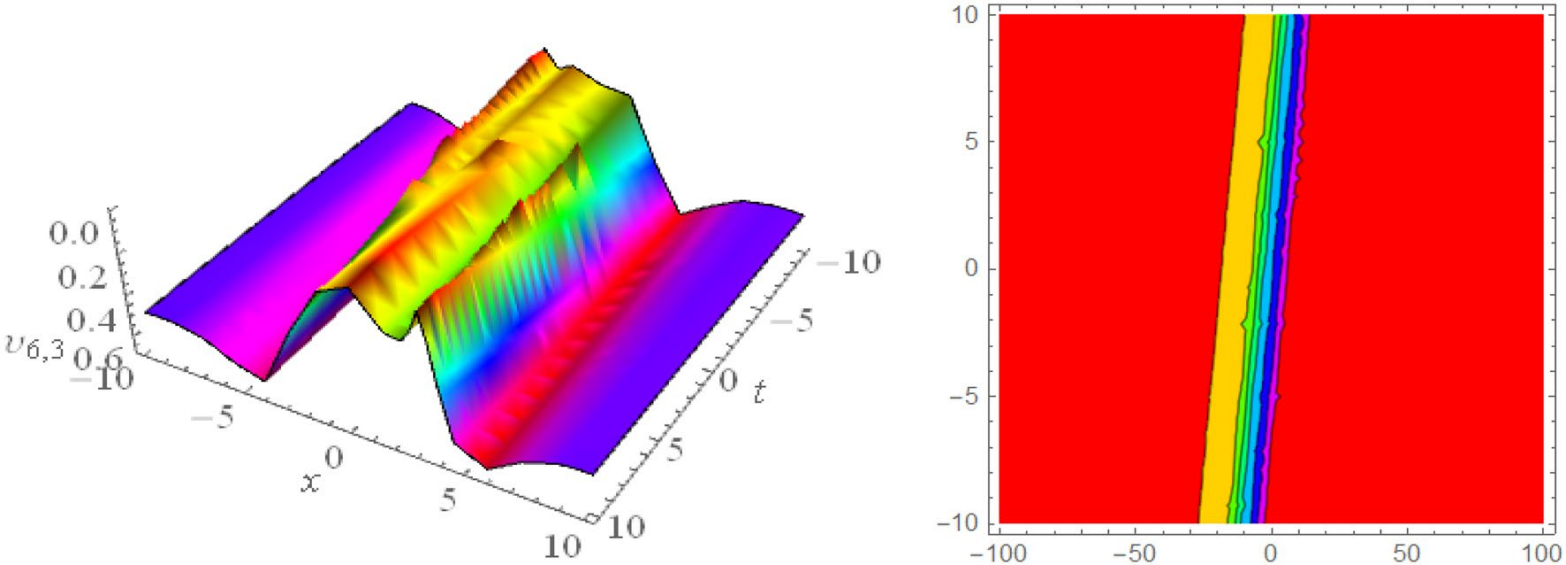
3D and corresponding contour for solution $\upsilon _{6,3}(x,t)$ with   $d_1=0.1,~~d_2=0.62,~~d_6=0.03,~~d_8=0.02,~~m_1=0.05,~~v=4.5,~~w_2=0.6,~~ w_3=0.5$.

In this section, we look at the graphical representations of the solutions we’ve found. The wave shapes depicted below are graphical representations of these solutions. We thoroughly investigate the behavior and distinguishing aspects of the solutions produced from the Heimburg equations through detailed explanations and accompanying graphs. These studies demonstrate the system’s amazing variety of waveforms, emphasizing its potential uses in biomembranes and nerves. Understanding the transmission of electrical impulses and the dynamics of bio-membrane structures in the context of the Heimburg model for biomembranes and nerves requires an understanding of breather waves, lump solitons, mixed multiwave solutions and other interactions. These are nonlinear wave solutions that are localised and oscillate amplitude and width on a periodic basis without changing their general shape. The interaction of dispersion and nonlinearity in the membrane, which influences the passage of electrical signals across nerve fibres, might result in these waves. Their physical importance comes from our understanding of how energy moves through biological membranes and is modulated, which affects the communication and propagation of nerve signals. These solitons may be associated with certain membrane fluctuations or structures, contributing to both signal transmission and membrane structural integrity. The importance is in comprehending how these localised structures remain stable and persistent in the intricate dynamics of biomembranes and neurons. The Figs. 1, 2, and 3 are drawn for the breather waves while Fig. 4 are the interaction of double exponents. The Figs. 5 and 6 clearly show the lump wave solutions. The Figs. 7, 8 and 9 give us the solitary waves by the mixed type solutions. Figure 10 also shows the limps in their interaction, while Figs. 11 and 12 explore the multiwaves. Figure 13 provided us with the dark soliton while Fig. 14 bright soliton. The Figs. 15, 16, 17 and 18 give us the periodic cross solution in their behaviors. These solutions are very attractive and helpful for the dynamic study of the Heimburg model and their interaction in medicine and the biological sciences.

## Conclusion

This study, used the Hirota Bilinear technique to explain soliton formation and propagation in biomembranes and nerves offers vital insights into the complex process of nerve impulse generation and transmission. This approach effectively found the exact travelling wave solutions to the Heimburg model of neurology, revealing innovative and different features such as kink, homoclinic wave, lump, mixed wave, multi wave and periodic-wave solutions. The significance of these results cannot be underestimated, because they offer insight into one of the most exciting issues in current biophysics-the basic mechanism that underpins life itself, the nerve impulse. This study has enhanced not just neurophysiology, but also mathematical physics as well. Furthermore, the graphical representations of the travelling wave solutions suggest the obtained unique profiles modulate into pulse patterns as they propagate through the axon. This dynamic behaviour gives crucial information for precisely understanding and managing the nerve impulse magnitude. The solutions obtained in this study are very remarkable because they have not being found in previous studies. The use of Mathematica 11.1 to validate these facts strengthens their accuracy and dependability. In the future, these analytic representations of solitary solutions offer possibilities for prospective applications in medicine and biosciences. They could be a useful tool for precise regulation of pulse magnitudes in nerve conduction, allowing for more investigation and breakthroughs in the understanding and treatment of many neurological disorders. Wave propagation issues in biomembranes and neurons, on the other hand, remain fascinating and challenging. More research is needed to expand on the foundation established by this study and other similar studies, and move further into the complexities surrounding this phenomena. In conclusion, the use of the Hirota Bilinear Method has made a substantial contribution to understanding the complexities of soliton formation and propagation in biomembranes and neurons. The findings of this study provide hope for both fundamental scientific understanding and future practical applications in the realms of medicine and bioscience. As we seek to investigate the intriguing issues of wave propagation in biomembranes, we go on an investigation to gain a better understanding of life’s fundamental processes. For the future work this study is helpful to obtained the different types of soliton solutions for the NLPDEs while the bilinear residual network method^14–18^ is also powerful technique to obtained the exact solutions for the integrable differential equations.

### Ethical approval

All the authors demonstrating that they have adhered to the accepted ethical standards of a genuine research study.

### Consent to participate

Being the corresponding author, I have consent to participate of all the authors in this research work.

## Data Availability

Data will be provided on request to the corresponding author.
